# Supplementary material for: Antibiotic-induced changes in the human gut microbiota for the most commonly prescribed antibiotics in primary care in the UK: a systematic review
Source: BMJ Open. 2020 Sep 21;10(9):e035677. doi: 10.1136/bmjopen-2019-035677 (PMC7507860; doi:10.1136/bmjopen-2019-035677)
Supplement: Author's manuscript [file bmjopen-2019-035677.draft_revisions.pdf]

BMJ Open

BMJ Open is committed to open peer review. As part of this commitment we make the peer review history of every article we publish publicly available.

When an article is published we post the peer reviewers' comments and the authors' responses online. We also post the versions of the paper that were used during peer review. These are the versions that the peer review comments apply to.

The versions of the paper that follow are the versions that were submitted during the peer review process. They are not the versions of record or the final published versions. They should not be cited or distributed as the published version of this manuscript.

BMJ Open is an open access journal and the full, final, typeset and author-corrected version of record of the manuscript is available on our site with no access controls, subscription charges or pay-per-view fees (http://bmjopen.bmj.com).

If you have any questions on BMJ Open's open peer review process please email info.bmjopen@bmj.com

BMJ Open

Antibiotic-induced changes in the human gut microbiota for the most commonly prescribed antibiotics in primary care in the United Kingdom: systematic review

Journal:	*BMJ Open*
Manuscript ID	bmjopen-2019-035677
Article Type:	Original research
Date Submitted by the Author:	21-Nov-2019
Complete List of Authors:	Elvers, Karen; University of Bristol, Bristol Medical School<br>Wilson, Victoria; University of Bristol, Bristol Medical School<br>Hammond, Ashley; University of Bristol, Bristol Medical School<br>Duncan, Lorna; University of Bristol, Bristol Medical School<br>Huntley, Alyson; University of Bristol, Bristol Medical School<br>Hay, Alastair; University of Bristol, Bristol Medical School<br>van der Werf, Esther; University of Bristol, Bristol Medical School; Louis Bolk Instituut, Health and Nutrition
Keywords:	PRIMARY CARE, Urinary tract infections < UROLOGY, Respiratory infections < THORACIC MEDICINE, Microbiology < PATHOLOGY

SCHOLARONE™
Manuscripts

I, the Submitting Author has the right to grant and does grant on behalf of all authors of the Work (as defined in the below author licence), an exclusive licence and/or a non-exclusive licence for contributions from authors who are: i) UK Crown employees; ii) where BMJ has agreed a CC-BY licence shall apply, and/or iii) in accordance with the terms applicable for US Federal Government officers or employees acting as part of their official duties; on a worldwide, perpetual, irrevocable, royalty-free basis to BMJ Publishing Group Ltd ("BMJ") its licensees and where the relevant Journal is co-owned by BMJ to the co-owners of the Journal, to publish the Work in this journal and any other BMJ products and to exploit all rights, as set out in our licence.

The Submitting Author accepts and understands that any supply made under these terms is made by BMJ to the Submitting Author unless you are acting as an employee on behalf of your employer or a postgraduate student of an affiliated institution which is paying any applicable article publishing charge ("APC") for Open Access articles. Where the Submitting Author wishes to make the Work available on an Open Access basis (and intends to pay the relevant APC), the terms of reuse of such Open Access shall be governed by a Creative Commons licence – details of these licences and which Creative Commons licence will apply to this Work are set out in our licence referred to above.

Other than as permitted in any relevant BMJ Author's Self Archiving Policies, I confirm this Work has not been accepted for publication elsewhere, is not being considered for publication elsewhere and does not duplicate material already published. I confirm all authors consent to publication of this Work and authorise the granting of this licence.

For peer review only

Antibiotic-induced changes in the human gut microbiota for the most commonly prescribed antibiotics in primary care in the United Kingdom: systematic review

For peer review only

Karen T Elvers, senior research associate[1], Victoria J Wilson, senior research associate (0000-0002-1657-3861)[1], Ashley Hammond, senior research associate[1], Lorna Duncan, senior research associate[1], Alyson Huntley, senior research fellow (0000-0001-9409-7891) [1], Alastair D Hay, professor of primary care (0000-0003-3012-375X)[1], Esther T van der Werf, senior lecturer in epidemiology (0000-0002-5330-5735)[1, 2]

[1]Centre for Academic Primary Care, Population Health Sciences, Bristol Medical School, University of Bristol, Canynge Hall, 39 Whatley Road, Bristol, BS8 2PS

[2]Louis Bolk Instituut, Kosterijland 3-5, 3981 AJ, Bunnik, The Netherlands

Corresponding author: Esther T. van der Werf, Tel: +31 6 86877696

e.vanderwerf@louisbolk.nl

Karen T Elvers karen.elvers@bristol.ac.uk

Victoria J Wilson victoria.wilson@bristol.ac.uk

Ashley Hammond ashley.hammond@bristol.ac.uk

Lorna Duncan lorna.duncan@bristol.ac.uk

Alyson Huntley alyson.huntley@bristol.ac.uk

Alastair D Hay alastair.hay@bristol.ac.uk

Short running title: The human gut microbiota and commonly prescribed antibiotics

For peer review only

Abstract

Objective The gut microbiota influences human health including digestion, energy metabolism and inflammation and its disruption has been linked to conditions such as inflammatory bowel disease and obesity. We investigated magnitude and duration of changes in gut microbiota in response to antibiotics commonly prescribed in primary care in the UK.

Methods We searched online databases to October 2018 including all study designs, collecting and analysing data on the effect of antibiotics prescribed for respiratory and urinary tract infections.

We used standard methods in accordance with PRISMA and Cochrane. Risk of bias was evaluated using the Critical Appraisal Skills Programme.

Main outcome measures Primary outcomes were antibiotic-induced changes in the composition and/or diversity of the gut microbiota. Secondary outcome was the time for the microbiota to return to baseline for the study population.

Results Twenty three included articles with low or unclear risk of bias showed antibiotics have an impact on the gut microbiota causing rapid and diminished levels of bacterial diversity and changes in relative abundances. After cessation of treatment, there was evidence that gut bacteria recover, in most individuals, to their baseline state within a few weeks, although some studies suggested longer term effects. Doxycycline was associated with a marked short-term decrease in *Bifidobacterium* diversity. Clarithromycin decreased the populations of Enterobacteria, and the anaerobic bacteria *Bifidobacterium* sp. and *Lactobacillus* sp. in numbers and diversity for up to 5 weeks. Phenoxymethylpenicillin, Nitrofurantoin and Amoxicillin had very little effect on the gut microbiome.

For peer review only

Conclusions Despite substantial heterogeneity, there is evidence that antibiotics commonly used in primary care influence the composition of the gastrointestinal microbiota. Research is limited, to mostly small populations. Larger population-based studies are needed to fully understand how the antibiotics modulate the microbiota, and to determine if these are associated with health consequences. Review registration number CRD42017073750 http://www.crd.york.ac.uk/PROSPERO

For peer review only

Strengths and limitations of this study

- This systematic review, to our knowledge, represents a comprehensive examination of antibiotic-induced changes in gut microbiota for the most commonly prescribed antibiotics in primary care in the UK for respiratory tract infections and urinary tract infections.
- Twenty-three studies were identified as meeting the inclusion criteria, with unclear risk of bias. The results showed that despite substantial heterogeneity, there is evidence that antibiotics impact on the gut microbiota causing rapid and diminished levels of bacterial diversity and changes in relative abundances. In most individuals, the microbiota recover to their baseline state within a few weeks of cessation of antibiotic treatment.
- The primary limitation was that the majority of the studies were small, and poorly randomised. There was considerable heterogeneity in methodology which is prone to biases and confounding factors.
- Larger population-based studies measuring the effect of antibiotics on the gut are required in order to make recommendations to clinicians.

For peer review only

Introduction

In the UK, between 2000 and 2014, antibiotic prescriptions increased from 14.3 to 19.7 defined daily doses (DDDs) per 1,000 inhabitants per day. The volume has decreased reaching 18.7 DDDs per 1,000 inhabitants per day in 2016 (http://www.oecd.org/). Misuse and overuse of antimicrobials, not only in medical sectors, but also veterinary and agricultural, results in increasing resistance which globally limits our ability to control infections. Decreasing antibiotic consumption is a key target to dealing with the problem and the UK has implemented antimicrobial stewardship interventions in primary care[1] and detailed a five year action plan for tackling antimicrobial resistance.[2]

Data analysed from The Health Improvement Network (THIN) database, stated a median prescribing rate among participating practices was 626 antibiotic prescriptions/1000 patients (IQR 543-699), 69% of which could be linked to a body system and/or clinical condition.[3] Of these prescriptions, 46% were linked to respiratory tract infections and 22.7% to infections of the urogenital tract. About half of all antibiotics prescribed were penicillin, of which approximately 55% was amoxicillin. This is followed by 13% macrolide use and 12% tetracycline.[3] General practice consultation rates in England and Wales show that a quarter of the population will visit their GP because of a respiratory tract infection each year.[4] Antibiotic use to treat such infection varies between individual clinicians and countries,[4-6] but accounts for 60% of all antibiotic prescribing in general practice.[7-9] Similarly, urinary tract infections are commonly seen in primary care.[10] Patients are frequently treated with antibiotics, even though the infection often is not microbiologically confirmed.[11] The overuse of antibiotics for conditions that are not serious or where antibiotic treatment is not appropriate for the illness, has led to their reduced effectiveness and emergence of resistant bacteria.

For peer review only

The normal gut microbiota consists of approximately 800–1000 different bacterial species and greater than 7000 different strains.[12] Normal is defined as the commensal species predominantly and consistently found in the gut of healthy human. The composition of the oesophageal and gastric microbiomes is distinct from that of the colon. There is a greater diversity of species and number per gram of contents in the colon, reflecting its higher pH.[13 14] The core microbiome in healthy individuals is dominated by phyla Firmicutes and Bacteroidetes (over 90%), followed by Verrucomicrobia and Actinobacteria.[13] Some essential functions of a healthy gut microbiota include vitamin production, nutrient metabolism and, protection against infection by inhibiting colonisation by pathogens and immunomodulation. Studies in gut microbiomes from different countries have identified differences in diversity which reflect the differences in diet, geography, early-life exposures and genetics.[15] Age, diet, antibiotics, probiotics and prebiotics all contribute to the shaping of the healthy gut microbiota and this continues dynamically throughout life.[13 14 16]

This leads to microbiome dysbiosis with increasing evidence that alterations to the human microbiome can affect how well the immune system functions and its ability to resist infection,[17 18] increase the risk of developing Parkinson's disease, multiple sclerosis and Alzheimer's disease,[19] the risk of depression, anxiety, or psychosis[20] and indirectly affect the health long term and risk of obesity and diabetes.[21] There is increasing evidence that the microbiome is crucial in a cancer patient's risk of infectious complications.[22] Recent studies have shown that antibiotic exposure in childhood is associated with increased risk for several diseases including obesity, types 1 and 2 diabetes, inflammatory bowel diseases, Crohn's disease, coeliac disease, autoimmune diseases such as allergies, juvenile idiopathic arthritis and asthma.[23-26] It appears that these effects are most pronounced if the antibiotics are

For peer review only

consumed within the first two years of life and the effects may well be cumulative.[17 27 28] This is particularly important in young children, when the adult microbiome has not been fully established.[29 30] Once antibiotic treatment has stopped, the microbiome may present a certain degree of resilience, being capable of returning to a composition similar to the original one, but this can take months or even years.[17 31]

Aim

To investigate the antibiotic-induced changes in gut microbiota for the most commonly prescribed antibiotics in primary care in the UK for respiratory tract infections and urinary tract infections. Our primary outcome was defining any antibiotic-induced changes in the composition of the gut microbiome, measured by 1) abundance and/or 2) diversity of the microbes, whilst our secondary outcome measured the time that is needed to restore the microbiome to a pre-antibiotic state.

Methods

A systematic literature review of studies that evaluated association between antibiotics commonly prescribed for respiratory tract and urinary tract infections in primary care and their effect on the gut microbiome was conducted. Preferred Reporting Items for Systematic Reviews and Meta-analyses (PRISMA) guidelines and checklist were used as a framework for this review (Table S1 PRISMA checklist).[32] The study protocol was registered with PROSPERO number, CRD42017073750 http://www.crd.york.ac.uk/PROSPERO.

Search Strategy

Electronic databases MEDLINE, EMBASE and AMED, were used to identify relevant articles up to October 2018, in English available as full text. A combination of search terms included the

For peer review only

following keywords: (antibacterial agents OR bacterial infections) AND (microbiota OR microbiome). The strategy was designed to identify studies conducted in any country, investigating antibiotic-induced changes in the human gut microbiota; analysed at either the individual or population-level, published in any language (Table 1). Reference lists of included studies and relevant reviews were hand searched to identify additional eligible studies. All selected abstracts and citations were exported from the scientific databases to the reference management software EndNote X9 (Thompson Reuters, New York, NY, USA) and duplicates excluded.

Study selection and eligibility criteria

A study was eligible for review if it reported quantitative changes to the gut microbiota due to treatment with antibiotics most commonly prescribed for respiratory and urinary tract infections prescribed in primary care (Table 2). Respiratory tract infections included both upper respiratory tract infections (URTIs), including the common cold, laryngitis, pharyngitis/tonsillitis, acute rhinitis, acute rhinosinusitis and acute otitis media and lower respiratory tract infections (LRTIs), including acute bronchitis, bronchiolitis, pneumonia and tracheitis. We included uncomplicated and recurrent urinary tract infections (UTI and rUTIs). We excluded any animal studies or hospital-based studies.

The main exposure of interest in this review was the effect commonly prescribed antibiotics have on the gut microbiome based on prescriptions advice included in the NICE guidelines [1 8 33-35] and on current use as described in nation reports on antibiotic use.[1 34] Of particular interest are those antibiotics, which are commonly used in primary care for the treatment of both upper and lower RTIs (amoxicillin, phenoxymethylpenicillin, doxycycline, co-amoxiclav, erythromycin, clarithromycin) and UTIs (trimethoprim, nitrofurantoin, amoxicillin, cephalexin).

For peer review only

169 **Screening, data extraction and management**

170 Five reviewers (VW, KE, EvdW, LD and AH) independently screened search results and
171 assessed each potential study according to the inclusion and exclusion criteria. All decisions
172 were recorded on a spreadsheet. Full text papers for all eligible studies were obtained and
173 three reviewers (VW, KE, AH) independently screened the selected papers a second time.

174 The following data were extracted from the included studies in an Excel spreadsheet by one
175 reviewer (KE) and a percentage verified for accuracy by another (VW): author, journal, year
176 of publication, study design, study country, number of participants, number of participants
177 of interest to this review, age range, percentage of female participants, other inclusion
178 criteria, exclusion criteria, recruitment methods, recruitment time period, sample type,
179 primary outcome, secondary outcome, antibiotic (of interest to this review) used, any other
180 antibiotics used, dose of antibiotics (of antibiotic of interest to this review), time course (of
181 antibiotic of interest to this review), number of timepoints for collecting samples, time
182 points for each sample collection, testing method, infection or condition, overall conclusion.
183 A tabular summary was developed for this review, which included study ID, country, design,
184 population, setting, infection, antibiotic of interest, sample and analysis, primary outcome
185 and main results (Table 3).

186 **Assessment of methodological quality**

187 Selection bias was assessed with the Critical Appraisal Skills Programme (CASP) checklist
188 (www.casp-uk.net). Each component of the studies (i.e., the appropriateness of the study
189 design for the research question, the risk of selection bias, exposure measurement, and
190 outcome assessment) was graded and an overall grading used to produce quality assessment
191 charts based on a traffic light system of ‘low’, ‘high’ and ‘unclear’ or ‘not applicable’, as

For peer review only

recommended by Cochrane.[36] Three reviewers (VW, KE, EvdW) independently assessed the risk of bias in the studies with one reviewer (KE) assessing all studies, and discrepancies were resolved by consensus between the reviewers.

Data synthesis and analysis

Due to the insufficient number of studies available, we were unable to undertake any sensitivity analysis to determine robustness of the findings and the quality of the studies.[36] The results are discussed descriptively.

Results

Overall

The database searches returned 1742 articles (October 2018). Following removal of duplicates and screening of records by title or abstract, 1487 were excluded with reasons detailed in Figure 1, based on our selection criteria. One hundred and twenty one full-text articles were evaluated and 23 were eligible and included in the present review. The included studies are summarised in Table 3 and are briefly described below.[31 37-57] Eight of the studies were published by different authors from the same Swedish study group.[38-41 43-45 50]

Publication dates ranged from 1991 to 2016. The studies measured the effects on gut microbiota following clinical treatment of the patient or experimental exposure of volunteers to different antimicrobial agents.

The 23 publications involved 885 patients, although not all of these received the antibiotics of interest and some were comparative control groups. Most studies originated from European countries, Sweden (7), Finland (3), Netherlands (2) and one each for France,

For peer review only

Germany, Switzerland, Belgium/Poland and Greece. The remaining studies were conducted in the USA (4), Chile (1) and Brazil (1).

Study Characteristics

Sampling

Faecal samples have generally been accepted as representative of the intestinal microbiota. Studies conducted before 2000, cultured homogenised samples on non-selective and selective media, enumerating different colony types. Some isolated pure cultures for identification to genus level. Their numbers were compared with the numbers of bacteria present from each genus before treatment to measure the effect of the antibiotic in decreasing the number of bacteria or allowing the increase, overgrowth or proliferation of others. However, it is known that many gut bacteria have not been cultured, about 80%.[58] Predominantly, later studies used more rapid molecular methods.

Analytical techniques

All analysed faecal samples, either by culture (12 studies) or molecular techniques (10 studies), one study used both methods. Ten of the studies used healthy volunteers, 4 of the studies patients had a respiratory infection, 3 a urinary tract infection, 1 reported urinary and respiratory, 1 sinusitis, 1 periodontitis, 1 relied on self-reported infections and 2 did not specify the health status of the subjects. Eight of the studies were from the same Swedish research institute (although one was conducted in Germany).

Risk of Bias

The risk of bias among the studies was assessed using the Critical Appraisal Skills Programme (CASP) checklists for case controls, RCTs (trials) and cohort studies (Figure 2). There was 1

case control appraisal, 5 cohort studies and 17 trials. All the studies adequately stated their study aims and hypothesis and this part of the assessment was rated as 'low' risk.

Case control

There was 1 case control study included in our review of a patient presenting with onset of diarrhoea not associated with Clostridium infection, within 24 h of antibiotic administration. [57] It was shown that this was associated with changes in the diversity of the gut microbiota. In terms of bias, the study trends towards overall unclear risk as there is lack of controls and the report details just one patient in a clinical setting with a symptom of interest (Figure 2a). However, they did use very robust molecular methods to study these temporal changes in the diversity of the microbiota.

Cohorts

There were five cohort studies included in our review. Assessment relating to the validity of the study are covered within the questions 1-6 (Figure 2b). Generally, these studies were low risk. The reliability of the studies determined by the size of the effect and how precisely this was estimated are covered with questions 7-9 and are low or unclear risk. There is greater unclear risk when it comes to the applicability of the results (questions 10-12), the usefulness for clinical decision making, often the population size was too small to extrapolate results to a larger population as a whole.

Trials

The majority of the studies included in this review fell in the category of trials/RCTs and overall the risk was more unclear. Concerning the validity of the studies (Q1-6) randomisation and blinding of treatment allocation was low risk in 65 and 47% of studies, respectively. Thus,

For peer review only

more than half of the studies were not blinded. Follow up of study participants was low risk, with most studies taking samples up to 4 weeks after antibiotic administration (although some indications this needs to be longer). The results (size and precision; Q7 and 8) and transferability of the results to other populations (Q9-11) were unclear risk.

Antibiotic effects on the gut

The studies analysed, employed different methodologies to explore the effect of antibiotics on the gut microbiota. They have been consistent in demonstrating that dysbiosis develops upon antibiotic administration, which is rapid and results in losses of diversity and shifts in abundance of gut microbes. After antibiotic treatment, the composition generally returns to a similar pre-treatment state within several weeks, but not in all cases. Here we describe the results in more detail per antibiotic of interest. There were no studies that reported on microbiota changes upon administration of erythromycin.

Amoxicillin

Nine studies examined the impact of orally administered amoxicillin on the normal intestinal microflora and are summarised in Table 4. In brief, administration of amoxicillin leads to an increase or over growth of Enterobacteria (5 studies). The changes in the anaerobic population (includes Lactobacillus, Bifidobacteria and Bacteriodes) varied enormously between studies: from no change, to increases and/or decreases, as well as changes in diversity. There were two reports that isolated Clostridia and Candida and two where no change in numbers were observed. Most studies reported that the populations returned to normal within 2-4 weeks, although some longer-term effects were seen, such as persistent Candida at 6 weeks, other aerobic or resistant Gram negative bacteria (species not specified)

For peer review only

at 28 days, persistent decrease in *Lachnospiraceae* at 6 months, with no similarities in the populations treated.

Christensson *et al.* [41] in a culture dependent randomised study of 84 subjects with lower respiratory tract infections, 38 of whom completed treatment with amoxicillin, showed that the numbers of enterobacteria, anaerobic Gram-positive Lactobacillus, Bifidobacteria and Eubacteria, and anaerobic Bacteroides increased significantly. The number of anaerobic cocci was reduced in 18 patients and increased in 11 patients. Four patients became colonised with *Pseudomonas* species and five with *C. albicans*. Although *C. albican*s was also isolated from two patients pre-treatment and three patients six weeks after treatment. *C. difficile* was isolated from two patients during treatment.

For peer review only

The effect of amoxicillin on the intestinal microflora of patients with bronchitis was evaluated in a randomised study by Floor *et al.* [47]. The anaerobic flora remained unchanged, both during, and after therapy with amoxicillin. The total number of aerobic Gram-negative rods increased significantly and in 37.5% of the patients with newly acquired bacteria the main species identified were *Klebsiella*. Enterococci were only cultured in 1 patient. A third of patients had other aerobic bacteria different to baseline on days 21-28.

Mangin *et al.* [51] studied 18 month old children with acute bronchitis. Both total bacteria and numbers of Bifidobacteria were similar during the course of antibiotic treatment. However, changes in Bifidobacterium at the species level showed decreased diversity. Amoxicillin treatment induced a complete disappearance of *Bifidobacterium adolescentis* species, a significant decrease in the occurrence rate of *Bifidobacterium bifidum*, but did not affect *Bifidobacterium longum* and *Bifidobacterium pseudocatenulatum/Bifidobacterium catenulatum*.

Monreal *et al.* [54] used culture based methods to examine the populations of faecal bacteria in 22 patients with respiratory tract infections treated with amoxicillin. *Bacteroides* spp., *Bifidobacterium* spp. and *Lactobacillus* spp. were decreased with antibiotic treatment. However, this was only transient and thirty days after the end of treatment the levels of Bifidobacterium and Lactobacillus had recovered to their normal values. Bacteroides were slower to recover.

Ladirat *et al.* [49] studied a small number of healthy volunteers with amoxicillin and a prebiotic specifically looking at total bacteria and Bifidobacterium. Numbers of Bifidobacterium decreased over time due to amoxicillin treatment especially in the group with no prebiotic. Amoxicillin affected the abundance and diversity of *Bifidiobacterium* spp. and there was an overgrowth of Enterobacteriaceae. The observations were highly individual dependent. In the follow-up period (3 weeks after the discontinuation of amoxicillin treatment), the levels of bifidobacterial in faeces returned to the initial levels.

Amoxicillin was administered randomly to a small number of healthy volunteers and the impact on intestinal microflora (and oropharyngeal) was studied by.[40] In the aerobic intestinal flora, a minor decrease in the numbers of streptococci and staphylococci was observed. Overgrowth of Klebsiella species was seen in six subjects and of Enterobacter species in two subjects. No overgrowth of enterococci or yeasts occurred. In the anaerobic microflora only the number of eubacteria was reduced. There was no colonisation by *C. difficile*. Two weeks after administration of amoxicillin the microbiota had returned to baseline levels.

Another study on the effect of amoxicillin on intestinal microflora of healthy volunteers showed only small alterations in the aerobic faecal microflora.[45] All major anaerobic bacterial groups were unaffected by amoxicillin and there was no colonisation by *C. difficile*. There was

For peer review only

and overgrowth of *Klebsiella* spp. in 6 subjects and Enterobacter in 2 subjects. Intestinal microflora returned to normal 2 weeks after treatment had been discontinued.

Six healthy volunteers received oral amoxicillin for 5 days and their faecal microbiota examined by 16S rRNA gene amplification and gradient gel electrophoresis until 60 days.[42] Dominant species diversity profiles were compared on the basis of similarity with percentages on first sampling ranging from 93% to 99%. During the 5 day course of amoxicillin treatment these percentages decreased to an average of 73%, but there was huge variation between individuals. By day 60, 5 of the 6 subject gel profiles had returned to their near initial composition. Banding patterns on the gels for 3 individuals were examined in more detail and were shown to correspond to *Clostridium nexile*, *Ruminococcus torques* and β-*Proteobacteria*.

Abeles *et al.* [37] studied cohabiting individuals over 6 months where one was given an antibiotic and the other a placebo. The microbiota of subjects taking amoxicillin grew more dissimilar over time although not statistically significant as similar trends were seen in those subjects taking placebo. Taxonomic compositions of the gut differed after amoxicillin therapy. *Lachnospiraceae* were significantly diminished and remained diminished at 6 months. Veillonellaceae, Bacteroidales, and Porphyromonadaceae were significantly decreased in response to amoxicillin, while Fusobacteriaceae were increased. Bifidobacteriales and Erysipelotrichaceae were initially decreased, and subsequently increased in comparison to their housemates taking placebo.

Amoxicillin with clavulanic acid

Five studies have looked at the impact of amoxicillin in combination with clavulanic acid on the gut microbiota.[31 46 48 50 57] These are summarised in Table 5. Briefly, the combination of

For peer review only

amoxicillin with clavulanic acid caused, mild to moderate changes in microbiome composition, mainly increases in Enterobacteria and fluctuations in anaerobic bacteria. No longer term effects were studied.

Administration of amoxicillin/clavulanic acid to healthy people caused increased numbers of enterococci and *E. coli* strains in the aerobic microflora while Bifidobacteria, Lactobacilli, and Clostridia decreased significantly.[50] Numbers of anaerobic cocci and Bacteriodes were not markedly altered. *C. difficile* strains were recovered from three volunteers. The microflora was normalised in all volunteers after 35 days.

Young and Schmidt[57] investigated the short-term impact of amoxicillin/clavulanic acid prescribed for a male patient with acute sinusitis, who developed antibiotic-associated diarrhoea. It was shown that the major bacterial groups were partially restored 14 days after antibiotic treatment, except for Bifidobacterium. During antibiotic administration no sequences corresponding to butyrate-producing *Clostridium* cluster XIVa were detected, but two weeks after cessation of antibiotics there was a reappearance of this cluster. In this case study the decrease in this cluster may be linked to the antibiotic-associated diarrhoea.

One arm of a study by Engelbrektson *et al.*[46] observed the effects of probiotic treatment concurrent with antibiotic therapy on faecal communities. They analysed their data by measuring individual divergences from baseline levels after treatment to address the problem of subject-to-subject variability. Subjects fell into two categories those with a stable baseline microbiota and those where it varied significantly. Culture data showed increasing numbers of Bacteroides and Enterobacteriaceae, but no trend for Clostridium, Bifidobacterium and Lactobacillus. The antibiotic group (without probiotic) had a significant change in numbers of bacteria from baseline at day 21.

Forssten *et al.*[48] also studied antibiotic administration in combination with probiotic. Consumption of the probiotic combination mainly lead to an increase in the faecal levels of the species included in the preparation. The antibiotic had only minor effects on Lactobacillus, Bifidobacterium, Bacteroides, Enterobacteriaceae and Clostridium.

Korpela *et al.*[31] looked a macrolides amoxicillin with and without clavulanic acid and penicillin V. Macrolide use reduced abundance of Actinobacteria and increased Bacteriodetes and Proteobacteria. Penicillin groups did not have distinctly different phyla composition. Firmicutes was reduced. The effect of macrolide use was long lasting and associated with increased risk of asthma and antibiotic-associated weight gain.

Nitrofurantoin

Three studies investigated the effect of nitrofurantoin macrocrystals on faecal microbiota compositions.[53 55] These are summarised in Table 3. Nitrofurantoin inhibits bacterial DNA, RNA, and cell wall protein synthesis. It is used prophylactically as a urinary anti-infective agent against most gram-positive and gram-negative organisms and for long-term suppression of infections. Upon oral administration, most of the nitrofurantoin is rapidly absorbed in the small intestine and eliminated primarily by kidney glomerular filtration into urine, where it reaches higher and more effective therapeutic concentrations. Only a small amount reaches the colon, which may account for the minor impact on intestinal microflora.

One of the studies used culture dependent techniques on a small population of women (n=7) with recurrent urinary tract infections.[53] Nitrofurantoin did not alter either the Enterobacteria, Enterococci or yeasts of the colonic flora during treatment. No resistant strains of Gram negative aerobic bacteria were detected.

For peer review only

Two later studies both used 16S rRNA PCR and sequencing. Stewardson *et al.*[59] treated UTI patients (n=10) with nitrofurantoin macrocrystals and showed that it was not associated with a statistically significant global impact on the gut microbiota (weak effect). Nitrofurantoin treatment was associated with an increase in the proportion of the genus Faecalibacterium;and decrease in the proportion Clostridium (Clostridiaceae) at the end of the antibiotic treatment. Another small study (n=8) treated uncomplicated UTI and did not show any significant impact of nitrofurantoin treatment on the faecal microbiota other than a temporary increase in the Actinobacteria phylum, more specifically in the beneficial Bifidobacterium genus.[55]

Doxycycline

Only two studies reported on the effect of doxycycline on faecal microbiota, one at sub optimal dosage (20mg for 9 months) in patients with periodontitis[56] and one at usual dose 150mg for 10 days but with a probiotic[52] as described in Table 3. Doxycycline treatment did not significantly affect counts of total anaerobic bacteria, candida, total enterics, Staphylococcus or doxycycline-resistant bacteria recovered at any of the sample periods and did not result in the development of multi-antibiotic resistance.[56] Matto *et al.*[52] specifically evaluated the influence of doxycycline therapy on the composition and antibiotic susceptibility of intestinal Bifidobacteria in nine subjects whilst they were also taking a probiotic and compared these to adults consuming only probiotics. A marked decrease in diversity of Bifidobacterium populations was observed during doxycycline therapy. Tetracycline-resistant Bifidobacterium isolates were more commonly detected in the antibiotic group than in the control group, thus increasing the pool of resistant commensal bacteria in the intestine.

For peer review only

416 In summary, doxycycline interferes with a microorganism's ability to manufacture proteins. At suboptimal dosage it has little effect on the gut microbiota. At normal dosage it markedly effects the diversity of Bifidobacteria populations. The effect on populations of other gut bacteria and long term effects are not known.

420 **Clarithromycin**

421 Three papers, from the same research institute, examined the intestinal microflora of healthy volunteers before, during and after administration of clarithromycin. The study design was similar in all three studies and involved small numbers, 10 (mix of male and female) in two studies[39 43] and 12 males only[44] as described in Table 3. Dosing and faecal sampling strategy and analysis were also similar, 250 mg twice daily for 7 days[39]; 500mg twice daily for 7 days[44] and 250 mg twice daily for 10 days,[43] sampling 3 times during and 3-4 times after administration up to 35 days. The male only subjects were administered another antibiotic with a 6 week wash out period, it is not clear whether this was before or after clarithromycin administration.

430 The main impact in the earlier study was a reduction in the numbers of Enterobacteria and Streptococci. Lactobacilli, Bifidobacteria and Bacteroides were suppressed in the anaerobic microflora.[39]

433 Clarithromycin caused a significant reduction of *E coli* during 7 days of treatment but levels returned to normal 28 days after the end of the course.[44] Six subjects were colonised by resistant aerobic Gram negative rods, Citrobacter, Klebsiella, Proteus and Pseudomonas. The total numbers of anaerobic bacteria decreased. Bifidobacteria and Bacteriodes were significantly reduced and Lactobacilli and clostridia were suppressed but this was not

For peer review only

significant. There was no overgrowth of *C. difficile* or yeasts. The microflora returned to normal in all subjects after 35 days.

In the final study, the numbers of *E. coli* were significantly reduced and in six individuals overgrowth of Klebsiella, Citrobacter and Enterobacter spp. occurred.[43] The total number of anaerobic intestinal bacteria was not affected but the numbers of Lactobacilli and Bifidobacteria were significantly reduced which persisted for the duration of the study. There was no significant overgrowth of *Candida* spp. and no subjects were colonised by *C. difficile*.

To summarise, all three studies reported reduction in Enterobacteria and suppression of anaerobic bacteria after administration of clarithromycin. This reduction was transient for most species except Lactobacilli and Bifidobacteria.

Phenoxymethylpenicillin

One study investigated the effect of phenoxymethylpenicillin on the oropharyngeal and intestinal microflora of healthy volunteers (Table 3).[38] No significant alterations in the total aerobic and anaerobic of the intestinal microflora were observed although three volunteers became newly colonised with *Klebsiella* sp. and one harboured high numbers of a non-fermentative gram-negative rod. The microflora became normalised 2 weeks after withdrawal of the drugs. The numbers of viridans streptococci, enterococci and Bacillus were unaffected by the administration of phenoxymethylpenicillin, while minor alterations were noticed in the numbers of *E. coli*. The median values of Clostridium species increased during administration, while the numbers of Bacteroides species were unaffected during the study period.

Discussion

For peer review only

This systematic review examined the use of antibiotics commonly prescribed in primary care to treat respiratory and urinary tract infections and their impact on gut microbiota. Firstly, the studies showed that antibiotics have an impact on the abundances of the bacteria in the gut community, causing rapid and diminished levels of bacterial diversity and taxonomic richness, increases and decreases in the relative abundances of certain taxa, leading to dysbiosis. As well as antibiotic- and individual host-specific effects.

Secondly, once treatment has stopped, there was some, but poor, evidence that the gut bacteria show resilience and are capable of some degree of recovery, in most individuals to their initial state. However, it is often not totally recovered, thus some antibiotics have a persistent effect on certain species. These observations underline the importance of restrictive and proper use of antibiotics in order to prevent long-term ecological disturbances of the indigenous microbiota.

Quantifying antibiotic therapy effect on gut flora is challenging. Although the literature describes 'normal' it really means those commensal species consistently and predominantly found in the human healthy gut. Studies quantifying changes in flora would be easier to interpret if a baseline population was established in each case. We found a lack of large-scale trials (RCTs) and observational studies and heterogeneity regarding methodology and outcomes. The heterogeneity is apparent by the inconsistency in the dosage, duration of treatment and follow up, selection and spectrum of antibiotics used, all contributing to varying faecal concentrations. The health status and participant age also have an effect, making comparative analysis impossible and therefore we have narratively described the effects observed.

The primary strength of this study is our focus on commonly prescribed antibiotics and their effect on gut microbiota. The information on antibiotics that most effect our gut microbiome

For peer review only

could influence GPs decision making in antibiotic prescribing in primary care. Also, the adverse impact of antibiotics on the gut microbiome is of great importance as when disrupted it can be associated with a variety of diseases, including infections, autoimmune diseases such as inflammatory bowel disease and diabetes, allergic disease and obesity. The main limitations are that the majority of the studies included were small, and not properly randomised, nor were they large observational studies. The considerable heterogeneity is prone to biases and confounding factors.

Inherent in many studies that address the impact of antibiotics on the intestinal microbiota are the limitations of techniques available for analysis. Many have been performed using laborious culture-based methodology which do not lend themselves to analysis of large numbers of samples. The older studies included in our analysis all used culture-based techniques to examine gut microbiota composition, which increases the risk of detection bias. Disadvantages of culturing are that despite the use of specific selective media and anaerobic incubation conditions, there remains a substantial part of the microbiota, approximately 80 %,[58] that has not yet been cultured. Culture media choice and sample handling can also skew the data.

The limitations of culture-based techniques can be largely overcome by using molecular approaches.[60] These methods are based on 16S rRNA gene amplification and give us a broader less biased view of gut bacterial composition. The amplified genes are characterised by methods, such as terminal-restriction fragment length polymorphism, denaturing or temperature gradient gel electrophoresis, deep shotgun metagenome and full-length sequencing, some of which are used in the more recent papers in our review. These bioinformatic techniques also have limitations in that they are often more difficult to understand and interpret. The use of different techniques makes it more difficult to compare

For peer review only

results and generalisation of conclusions is a result of this diversity in determination and quantification.

The composition of the gut microbiota among humans varies considerably from subject to subject. Grouping of data from several individuals can result in loss of statistical significance. Other external factors such as diet, stress or taking probiotic supplements can complicate the specific antibiotic treatment effects on the gut microbiome profile and these factors change over a lifetime.

Conclusions

The widespread and often overuse of antibiotics has led to the establishment of antibiotic-resistant bacteria and the transfer of resistance genes, which has become a global challenge for infection control and the untreatable nature of bacterial infections. Almost 80% of the antibiotics within the NHS are being prescribed in primary care[1]. The effects of excessive antibiotic exposure can also be seen in the symbiotic microbiotas of the human body.[61] As a result, the microbiota imbalances caused by antibiotics can negatively affect health by increasing susceptibility to infections, compromising immune homeostasis (indicative in increased allergies), asthma, IBS and deregulated metabolism seen by obesity, metabolic syndrome and diabetes.[62]

Antibiotics have an impact on the gut microbiota causing rapid and diminished levels of bacterial diversity and increases and decreases in the relative abundances, leading to dysbiosis. Once treatment has stopped, there was some evidence that the gut bacteria are capable of some degree of recovery, in most individuals, to their baseline state.

This systematic review highlights the lack of research and understanding of the effects of commonly prescribed antibiotics on the gut microbiome and shows it is an area which

For peer review only

requires studies on larger populations with extended sampling to understand the long-term impacts.

Footnotes

Contributors: KTE and VW performed the searches. VW, EvdW, AH, LD and KTE identified eligible studies. KTE, VW and EvdW authors appraised study quality and extracted data. KTE and VW drafted first sections of the text and KTE completed data analysis, text and tables to circulate to all authors for their contribution to the final draft. EvdW is the guarantor and attests that all listed authors meet authorship criteria and that no others meeting the criteria have been omitted. All authors had full access to the data in the study and can take responsibility for the integrity of the data and the accuracy of the data analysis.

Funding: This research was supported by the NIHR Health Protection Research Unit in Evaluation of Interventions at the University of Bristol. The views expressed in this article are those of the author(s) and not necessarily those of the NHS, the NIHR, or the Department of Health and Social Care.

Competing interests: All authors have completed the ICMJE's unified disclosure form at http://icmje.org/conflicts-of-interest/ and declare: all authors had financial support from the University of Bristol for the submitted work; no financial relationships with any organisations that might have an interest in the submitted work in the previous three years; no other relationships or activities that could appear to have influenced the submitted work."

Ethical approval: Not required.

Transparency declaration: Dr Esther van der Werf (the manuscript's guarantor) affirms that the manuscript is an honest, accurate, and transparent account of the study being reported; and that no important aspects of the study have been omitted.

For peer review only

554 **Copyright:** Dr Esther van der Werf has the right to grant on behalf of all authors and does
555 grant on behalf of all authors, an exclusive licence on a worldwide basis to the BMJ Publishing
556 Group Ltd to permit this article (if accepted) to be published in BMJ editions and any other
557 BMJPGL products and sublicences such use and exploit all subsidiary rights, as set out in our
558 licence.

559 **Patient and Public Involvement:** not applicable for this work.

560 **Data sharing:** if necessary data can be shared.

561

For peer review only

References

1. Public Health England. English surveillance programme for antimicrobial utilisation and resistance (ESPAUR) London: Public Health England, 2018:https://assets.publishing.service.gov.uk/government/uploads/system/uploads/attachment_data/file/759975/ESPAUR_2018_report.pdf.

2. Health Protection Agency. Tackling antimicrobial resistance 2019-2024: The UK's 5-year action plan, 2019:1-91.

3. Dolk FCK, Pouwels KB, Smith DRM, et al. Antibiotics in primary care in England: which antibiotics are prescribed and for which conditions? *J Antimicrob Chemoth* 2018;73(suppl_2):ii2-ii10. doi: 10.1093/jac/dkx504 %J Journal of Antimicrobial Chemotherapy

4. Ashworth M, Charlton J, Ballard K, et al. Variations in antibiotic prescribing and consultation rates for acute respiratory infection in UK general practices 1995-2000. *Brit J Gen Pract* 2005;55(517):603-08.

5. Stanton N, Francis NA, Butler CC. Reducing uncertainty in managing respiratory tract infections in primary care. *British Journal of General Practice* 2010;60(581):e466-75.

6. Howie JG. Clinical judgement and antibiotic use in general practice. *British Medical Journal* 1976;2(6043):1061-4.

7. Lindbaek M. Prescribing antibiotics to patients with acute cough and otitis media. *British Journal of General Practice* 2006;56(524):164-6.

8. Tan T, Little P, Stokes T. Antibiotic prescribing for self limiting respiratory tract infections in primary care: summary of NICE guidance. *BMJ* 2008;337

9. Juszczyk D, Charlton J, McDermott L, et al. Electronically delivered, multicomponent intervention to reduce unnecessary antibiotic prescribing for respiratory infections in primary care: a cluster randomised trial using electronic health records—REDUCE

For peer review only

587 Trial study original protocol. *BMJ Open* 2016;6(8) doi: 10.1136/bmjopen-2015-
588 010892
589 10. Car J. Urinary tract infections in women: diagnosis and management in primary care. *Bmj*
590 2006;332 doi: http://dx.doi.org/10.1136/bmj.332.7533.94
591 11. Mangin D. Urinary tract infection in primary care. *Bmj* 2010;340 doi: 10.1136/bmj.c657
592 12. The Human Microbiome Project C, Huttenhower C, Gevers D, et al. Structure, function
593 and diversity of the healthy human microbiome. *Nature* 2012;486:207. doi:
594 10.1038/nature11234https://www.nature.com/articles/nature11234#supplementar
595 y-information
596 13. Hollister EB, Gao C, Versalovic J. Compositional and functional features of the
597 gastrointestinal microbiome and their effects on human health. *Gastroenterology*
598 2014;146(6):1449-58. doi: 10.1053/j.gastro.2014.01.052 [published Online First:
599 2014/01/29]
600 14. Jandhyala SM, Talukdar R, Subramanyam C, et al. Role of the normal gut microbiota.
601 *World journal of gastroenterology* 2015;21(29):8787-803. doi:
602 10.3748/wjg.v21.i29.8787 [published Online First: 2015/08/07]
603 15. Lloyd-Price J, Abu-Ali G, Huttenhower CJGM. The healthy human microbiome.
604 2016;8(1):51. doi: 10.1186/s13073-016-0307-y
605 16. Tuddenham S, Sears CL. The intestinal microbiome and health. *Current opinion in*
606 *infectious diseases* 2015;28(5):464-70. doi: 10.1097/QCO.0000000000000196
607 17. Langdon A, Crook N, Dantas G. The effects of antibiotics on the microbiome throughout
608 development and alternative approaches for therapeutic modulation. *Genome*
609 *Medicine* 2016;8(1):39.

For peer review only

18. Denny JE, Powell WL, Schmidt NW. Local and Long-Distance Calling: Conversations between the Gut Microbiota and Intra- and Extra-Gastrointestinal Tract Infections. *Frontiers in Cellular & Infection Microbiology* 2016;6:41.

19. Tremlett H, Bauer KC, Appel-Cresswell S, et al. The gut microbiome in human neurological disease: A review. *Annals of Neurology* 2017;81(3):369-82. doi: 10.1002/ana.24901

20. Lurie I, Yang YX, Haynes K, et al. Antibiotic exposure and the risk for depression, anxiety, or psychosis: a nested case-control study. *Journal of Clinical Psychiatry* 2015;76(11):1522-8.

21. Cox LM, Blaser MJ. Antibiotics in early life and obesity. *Nature Reviews Endocrinology* 2015;11(3):182-90.

22. Taur Y, Pamer EG. Microbiome mediation of infections in the cancer setting. *Genome Medicine* 2016;8(1):40.

23. Blaser MJ. Antibiotic use and its consequences for the normal microbiome. *Science* 2016;352(6285):544-5.

24. Ahmad OF, Akbar A. Microbiome, antibiotics and irritable bowel syndrome. *British medical bulletin* 2016;120(1):91-99.

25. Mikkelsen KH, Allin KH, Knop FK. Effect of antibiotics on gut microbiota, glucose metabolism and body weight regulation: a review of the literature. *Diabetes, obesity & metabolism* 2016;18(5):444-53. doi: https://dx.doi.org/10.1111/dom.12637

26. Li M, Wang M, Donovan SM. Early development of the gut microbiome and immune-mediated childhood disorders. *Seminars in Reproductive Medicine* 2014;32(1):74-86.

27. Lankelma JM, Nieuwdorp M, de Vos WM, et al. The gut microbiota in internal medicine: implications for health and disease. *Netherlands Journal of Medicine* 2015;73(2):61-8.

For peer review only

28. Turta O, Rautava S. Antibiotics, obesity and the link to microbes - what are we doing to our children? *BMC Medicine* 2016;14:57.

29. Cassir N, Simeoni U, La Scola B. Gut microbiota and the pathogenesis of necrotizing enterocolitis in preterm neonates. *Future Microbiol* 2016;11(2):273-92. doi: 10.2217/fmb.15.136 [published Online First: 2016/02/09]

30. Yatsunenko T, Rey FE, Manary MJ, et al. Human gut microbiome viewed across age and geography. *Nature* 2012;486(7402):222-7. doi: 10.1038/nature11053 [published Online First: 2012/06/16]

31. Korpela K, Salonen A, Virta LJ, et al. Intestinal microbiome is related to lifetime antibiotic use in Finnish pre-school children. *Nature communications* 2016;7:10410.

32. Moher D, Liberati A, Tetzlaff J, et al. Preferred reporting items for systematic reviews and meta-analyses: the PRISMA statement. *PLoS Med* 2009;6(7):e1000097. doi: 10.1371/journal.pmed.1000097 [published Online First: 2009/07/22]

33. McDonald LC. Effects of short- and long-course antibiotics on the lower intestinal microbiome as they relate to traveller's diarrhea. *Journal of travel medicine* 2017;24(suppl_1):S35-S38. doi: https://dx.doi.org/10.1093/jtm/taw084

34. Duranti S, Lugli GA, Mancabelli L, et al. Prevalence of Antibiotic Resistance Genes among Human Gut-Derived Bifidobacteria. *Applied and environmental microbiology* 2017;83(3) doi: https://dx.doi.org/10.1128/AEM.02894-16

35. Excellence NIfHaC. Urinary tract infections in adults. London: NICE, 2015.

36. Higgins J, Green S. Cochrane Handbook for Systematic Reviews of Interventions Version 5.1.0 [updated March 2011]. *The Cochrane Collaboration* 2011

37. Abeles SR, Jones MB, Santiago-Rodriguez TM, et al. Microbial diversity in individuals and their household contacts following typical antibiotic courses. *Microbiome* 2016;4(1):39. doi: https://dx.doi.org/10.1186/s40168-016-0187-9

For peer review only

38. Adamsson I, Edlund C, Sjostedt S, et al. Comparative effects of cefadroxil and phenoxymethylpenicillin on the normal oropharyngeal and intestinal microflora. *Infection* 1997;25(3):154-8. [published Online First: 1997/05/01]

39. Brismar B, Edlund C, Nord CE. Comparative effects of clarithromycin and erythromycin on the normal intestinal microflora. *Scandinavian Journal of Infectious Diseases* 1991;23(5):635-42. doi: 10.3109/00365549109105189

40. Brismar B, Edlund C, Nord CE. Impact of cefpodoxime proxetil and amoxicillin on the normal oral and intestinal microflora. *European journal of clinical microbiology & infectious diseases : official publication of the European Society of Clinical Microbiology* 1993;12(9):714-9. [published Online First: 1993/09/01]

41. Christensson B, Nilsson-Ehle I, Ljungberg B, et al. A randomized multicenter trial to compare the influence of cefaclor and amoxycillin on the colonization resistance of the digestive tract in patients with lower respiratory tract infection. *Infection* 1991;19(4):208-15. doi: 10.1007/BF01644947

42. De La Cochetiere MF, Durand T, Lepage P, et al. Resilience of the dominant human fecal microbiota upon short-course antibiotic challenge. *Journal of Clinical Microbiology* 2005;43(11):5588-92.

43. Edlund C, Alvan G, Barkholt L, et al. Pharmacokinetics and comparative effects of telithromycin (HMR 3647) and clarithromycin on the oropharyngeal and intestinal microflora. *The Journal of antimicrobial chemotherapy* 2000;46(5):741-9. [published Online First: 2000/11/04]

44. Edlund C, Beyer G, Hiemer-Bau M, et al. Comparative effects of moxifloxacin and clarithromycin on the normal intestinal microflora. *Scandinavian Journal of Infectious Diseases* 2000;32(1):81-85.

For peer review only

45. Edlund C, Stark C, Nord CE. The relationship between an increase in beta-lactamase activity after oral administration of three new cephalosporins and protection against intestinal ecological disturbances. *The Journal of antimicrobial chemotherapy* 1994;34(1):127-38. [published Online First: 1994/07/01]

46. Engelbrektson A, Korzenik JR, Pittler A, et al. Probiotics to minimize the disruption of faecal microbiota in healthy subjects undergoing antibiotic therapy. *Journal of Medical Microbiology* 2009;58(Pt 5):663-70.

47. Floor M, Akkeren FV, Rozenberg-Arska M, et al. Effect of loracarbef and amoxicillin on the oropharyngeal and intestinal microflora of patients with bronchitis. *Scandinavian Journal of Infectious Diseases* 1994;26(2):191-97. doi: 10.3109/00365549409011783

48. Forssten S, Evans M, Wilson D, et al. Influence of a probiotic mixture on antibiotic induced microbiota disturbances. *World Journal of Gastroenterology* 2014;20(33):11878-85.

49. Ladirat SE, Schoterman MH, Rahaoui H, et al. Exploring the effects of galacto-oligosaccharides on the gut microbiota of healthy adults receiving amoxicillin treatment. *British Journal of Nutrition* 2014;112(4):536-46.

50. Lode H, Von der Hoh N, Ziege S, et al. Ecological effects of linezolid versus amoxicillin/clavulanic acid on the normal intestinal microflora. *Scand J Infect Dis* 2001;33(12):899-903. [published Online First: 2002/03/01]

51. Mangin I, Suau A, Gotteland M, et al. Amoxicillin treatment modifies the composition of Bifidobacterium species in infant intestinal microbiota. *Anaerobe* 2010;16(4):433-8. doi: 10.1016/j.anaerobe.2010.06.005 [published Online First: 2010/07/06]

52. Matto J, Maukonen J, Alakomi HL, et al. Influence of oral doxycycline therapy on the diversity and antibiotic susceptibility of human intestinal bifidobacterial population.

For peer review only

Journal of applied microbiology 2008;105(1):279-89. doi: 10.1111/j.1365-2672.2008.03792.x [published Online First: 2008/04/10]

53. Mavromanolakis E, Maraki S, Samonis G, et al. Effect of norfloxacin, trimethoprim-sulfamethoxazole and nitrofurantoin on fecal flora of women with recurrent urinary tract infections. *J CHEMOTHER* 1997;9(3):203-07. doi: 10.1179/joc.1997.9.3.203

54. Monreal MT, Pereira PC, de Magalhaes Lopes CA. Intestinal microbiota of patients with bacterial infection of the respiratory tract treated with amoxicillin. *Brazilian Journal of Infectious Diseases* 2005;9(4):292-300.

55. Vervoort J, Xavier BB, Stewardson A, et al. Metagenomic analysis of the impact of nitrofurantoin treatment on the human faecal microbiota. *The Journal of antimicrobial chemotherapy* 2015;70(7):1989-92. doi: 10.1093/jac/dkv062 [published Online First: 2015/03/15]

56. Walker C, Preshaw PM, Novak J, et al. Long-term treatment with sub-antimicrobial dose doxycycline has no antibacterial effect on intestinal flora. *Journal of Clinical Periodontology* 2005;32(11):1163-69.

57. Young VB, Schmidt TM. Antibiotic-associated diarrhea accompanied by large-scale alterations in the composition of the fecal microbiota. *Journal of Clinical Microbiology* 2004;42(3):1203-6.

58. Eckburg PB, Bik EM, Bernstein CN, et al. Diversity of the human intestinal microbial flora. *Science* 2005;308(5728):1635-8. doi: 10.1126/science.1110591 [published Online First: 2005/04/16]

59. Stewardson AJ, Gaia N, Francois P, et al. Collateral damage from oral ciprofloxacin versus nitrofurantoin in outpatients with urinary tract infections: a culture-free analysis of gut microbiota. *Clinical Microbiology & Infection* 2015;21(4):344.e1-11.

For peer review only

60. Brugère J-F, Mihajlovski A, Missaoui M, et al. Tools for stools: The challenge of assessing human intestinal microbiota using molecular diagnostics2009.

61. Francino MP, Moya A. EFFECTS OF ANTIBIOTIC USE ON THE MICROBIOTA OF THE GUT AND ASSOCIATED ALTERATIONS OF IMMUNITY AND METABOLISM. *EMJ Gastroenterol* 2013;1:74-80.

62. Sekirov I, Russell SL, Antunes LCM, et al. Gut Microbiota in Health and Disease. *Physiological Reviews* 2010;90(3):859-904. doi: 10.1152/physrev.00045.2009

For peer review only

Table 1. Search strategy

1. Anti-Bacterial Agents/ae, pd, tu
2. Bacterial Infections/dt, mi
3. Microbiota/
4. (Microbiome or Microbiomes or Gut microbiome or Gut microbiomes or Microbiota Gut or microbiota).mp.
5. Humans/
6. 1 or 2
7. 3 or 4
8. 6 and 7
9. 5 and 8

For peer review only

Table 2. PICOS criteria for inclusion and exclusion of studies.

**Parameter**	**Inclusion**	**Exclusion**
Participants	Humans adults and children	Animals, *in vitro*
Intervention	Specific antibiotics of interest prescribed in primary care	Other antibiotics
Comparator	Negative controls or other antibiotics	None
Outcomes	Changes in gut microbiota, total numbers, diversity and composition; time taken to recover	Antimicrobial resistance
Study design	All study types	None

For peer review only

Table 3. Summary of studies included in the review

Study ID	Country	Design	Population	Setting	Infection	Antibiotic of interest (and dosage)	Sample and analysis	Primary Outcome	Results
**Amoxicillin**									
**De La Cochetiere et al. 2005**	France	Not specified	N = 6 adults	Volunteers	Healthy	Amoxicillin (500mg three times a day for 5 days)	Faecal samples; 16S rRNA PCR and TTGE gel analysis and sequence analysis	To assess the ability of the human faecal microbiota to return to its original dominant species profile after a 5-day course of amoxicillin	Similarity indices of the TTGE profiles on D1 ranged from 93-99%; on D3 62-82% and D4 46-94%. On D30 and D60 these had raised back to 65-95% and 66-98% respectively. Bands excised from the gel related to *Clostridium nexile* and *Ruminococcus torques* (and β-Proteobacteria).
**Monreal et al. 2005**	Brazil	Prospective	N = 42 adults; 20 controls	Emergency department ; blood donor centres	Bacterial respiratory infections (sinusitis, pneumonia)	Amoxicillin	Faecal samples analysed by culture and CFU on blood agar	Investigated the influence of respiratory tract infections and of amoxicillin therapy on the normal intestinal microbiota of patients	Concentration of *Bacteriodes*, *Bifidobacterium* spp. *Lactobacillus* spp. were significantly decreased in the antibiotic group. After 30 days levels recovered to normal.

For peer review

**Abeles et al. 2016**	USA	Cohort of unrelated cohabiting individuals randomly assigned	N = 56 adults	University campus	Self reported health status	Amoxicillin 500 mg twice daily for 3 and 7 days	16S rRNA amplified from DNA using QIAGEN stool Mini Kit; sequencing	Effects of commonly prescribed antibiotics on microbiota	Microbiota grew more dissimilar over time but not significant. Most abundant taxa in gut were Bacteroidaceae, Lachnospiraceae and Ruminococcaceae. Lachnospiraceae, Veillonellaceae, Bacteroidales and Porphyromonadaceae were significantly decreased. Fusobacteriaceae increased. Bifidobacteriales and Erysipelotrichaecae initially decreased.
**Mangin et al. 2010**	Chile	Study trial	N = 42 infants; 31 with treatment	Health centre	Acute bronchitis	Amoxicillin 50 mg/kg/d in three daily doses for 7 days	Faecal; real time PCR for Bifidobacterium, TTGE and sequence analysis	to investigate the quantitative and qualitative changes occurring in the faecal bifidobacterial populations in 18 month children after a one-week amoxicillin treatment	Total bacteria and numbers of Bifidobacteria were not significantly altered by amoxicillin treatment. However, treatment changed species diversity, a complete disappearance of *B. adolescentis*, increase in *B. bifidum*. No effect on *B. longum*, *B. pseudocatenulatum*/*B. catenulatum*.
**Christensson et al. 1991**	Sweden	randomised double blind parallel	N = 84 adults; 44 with treatment (38 /44 completed)	outpatients	Lower respiratory tract infection	Amoxicillin 250 mg t.i.d for 7 days	Faecal; cultured aerobically and anaerobically in broth to ID	To compare cefaclor and amoxycillin as treatment for lower respiratory tract infections	Streptococci, staphylococci and enterococci were unchanged in numbers, while enterobacteria significantly increased. *Pseudomonas* sp. and *Candida albicans* were isolated from some samples. Anaerobic cocci numbers were reduced in some

		multicentre trial					pathogenic microorganism and on selective plates for analysis of microflora changes	and in their ability to influence colonization resistance	patients and increased in others. lactobacilli, bifidobacteria, bacteriodes and eubacteria increased. A couple of patients were colonised with *C. difficile*.
**Brismar et al. 1993**	Sweden	single blind randomised comparative study	N = 20 adults; 10 with treatment	Not specified	Healthy volunteers	Amoxicillin 500 mg every 8 h for 7 days	Faecal; selective media, MICs, beta lactamase activity	to study the oral and intestinal microflora before, during and after administration of cefpodoxime proxetil and amoxicillin	Numbers of aerobic intestinal microflora were slightly affected by amoxicillin administration. A minor decrease in numbers of streptococci and staphylococci, an over growth of Klebsiella and Enterobacter in a couple of samples. No overgrowth of enterococci or yeasts occurred. Eubacteria was reduced by amoxicillin. No colonisation by *C. difficile*. Flora returned to normal 2 weeks after administration of amoxicillin. Amoxicillin treatment strongly induced emergence of new colonizing amoxicillin resistant microorganisms in the normal faecal microflora, mainly *E. coli*, *Klebsiella* sp. and *Enterobacter* sp.
**Edlund et al. 1994**	Sweden	Not specified	N = 44 adults, 10 with treatment	Not specified	Not specified	Amoxicillin 500 mg t.i.d for 7 days	Faecal; serial dilutions and selective plating	The investigation was focused on drug concentrations in	Administration of amoxicillin affected the aerobic intestinal microflora to a minor extent. There was an overgrowth of *Klebsiella* spp. and *Enterobacter*

For peer review only - http://bmjopen.bmj.com/site/about/guidelines.xhtml

							aerobic and anaerobic	faeces, beta lactamase production by the intestinal microflora, alterations in the microflora and susceptibility patterns	spp in some samples. All major anaerobic bacterial groups remained unaffected during the investigation, no colonisation with *C. difficile*. Intestinal microflora returned to normal 2 weeks after treatment stopped.
**Ladirat et al. 2014**	Netherlands	double blinded randomised parallel intervention study	N = 12 adults (2 not analysed due to non compliance)	Not specified	Healthy volunteers	Amoxicillin 375mg three times daily for 5 days	Faecal; Intestinal (I)-Chip microarray, total bacteria and Bifidobacterium spp using qPCR	determined the effects of prebiotic intake on the microbiota of healthy adult subjects during and after treatment with amoxicillin	Total bacteria and Bifidobacterium were similar in both groups before treatment. Numbers of Bifidobacterium decreased over time due to amoxicillin treatment in both groups, but more so in the antibiotic only group (no prebiotic). After antibiotic treatment numbers in the antibiotic only group were still lower than with prebiotics. Abundance of *Bifidobacterium* spp. *B. longum* and *B. thermophilum* was significantly higher in the prebiotic group. The composition of the microbiota in most subjects returned to normal but there were higher Bifidobacterium in the prebiotic group. Due to amoxicillin treatment, a decrease in the abundance of *Bifidobacterium* spp., an overgrowth of Enterobacteriaceae

For peer review only - http://bmjopen.bmj.com/site/about/guidelines.xhtml

**Floor et al. 1994**	Netherlands	randomised double blind study	N = 80 adults	recruited from 9 General Practices	chronic bronchitis with purulent sputum	Amoxicillin, 500 mg 3 times a day for 7 days	Faecal; cultured aerobically and anaerobically on selective media, testing for C. difficile, enterococci, staphylococci, yeasts and biochemical identification	effect of both amoxicillin and loracarbef on oropharyngeal and intestinal microflora	Total number of anaerobic bacteria remained unchanged both during and after therapy with amoxicillin. Total numbers of aerobic Gram negative bacteria significantly higher on days 8-10 than at baseline. Enterococci were only cultured in 1 patient in amoxicillin group. Yeast numbers increased significantly but returned to baseline on days 21-28. Presence of other aerobic bacteria occurred in a slightly higher percentage of patients in the amoxicillin group. 13/15 new acquired bacteria in the amoxicillin group were Klebsiella. In about a third of patients, bacteria still present at 21-28 days. Amoxicillin treatment for bronchitis was 90% effective in amoxicillin group.
**Amoxicillin and Clavulanic acid**									
**Korpela et al. 2016**	Finland	Part of a cohort from larger probiotic trial	N = 236 children; 142 donated faecal samples	Day care centres	Antibiotic use for respiratory (88%) other mainly urinary (5%)	penicillins (amoxicillin with or without clavulanic acid and penicillin V), macrolides (azithromyci	Faecal by 16S rRNA and sequence analysis; operational taxonomic unit (OTU); culture based antibiotic	Use of phylogenetics, metagenomics and antibiotic use on microbiota composition and metabolism	Macrolide use reduced abundance of Actinobacteria and increased Bacteriodetes and Proteobacteria. Penicillin groups did not have distinctly different phyla. Firmicutes was reduced. The effect of macrolide use was long lasting and associated with increased risk of asthma and antibiotic-associated weight gain.

For peer review only

						n and clarithromycin) and sulphonamide–trimethoprim	sensitivity testing		
**Forssten et al. 2014**	Finland	Randomised double blind placebo controlled parallel study	N = 80 adults; 40:40 probiotic:placebo	Volunteers	Healthy	Augmentin (875mg Amoxicillin and 125 mg clavulanate) for 7 days	Faecal samples; qPCR for specific microbial groups	To investigate the effect of a specific combination of probiotic strains on the incidence of antibiotic induced microbiota disturbances	Generally Lactobacillus and Bifidobacterium were increased/restored in the probiotic group and reduced in the placebo group. Antibiotics reduced levels of Clostridium in both groups, but increased Enterobacteriaceae
**Young et al. 2004**	USA	Case report	N = 1 adult male	Not detailed	Acute sinusitis	amoxicillin-clavulanic acid (875 and 125 mg, respectively, twice daily for 10 days)	Faecal sample 16S rRNA PCR and sequencing and analysis	Molecular phylogenetic survey of the faecal microbiota from a patient who developed antibiotic associated diarrhoea during the administration of	D0- sequences clustered within 4 bacterial groups; *Bacteroides* spp., *Clostridium* sp IV, *Clostridium* sp XIVa and *Bifidobacterium*. At D4- *Bacteroides* still major component but *B. distasonis* group not *B. fragilis* cluster. No *Clostridium* sp. XIVa or *Bifidobacterium* spp. 34% were now *Enterobacteriaceae*. D24- partial reversal, *B. fragilis* predominated and cluster XIVa returned, no *Enterobacteriaceae*, no return of *Bifidobacterium* spp.

								a broad-spectrum antibiotic	
**Engelbrekston et al. 2009**	USA	probiotic-antibiotic study arm	N = 40 adults	Not specified	Healthy volunteers	Agumentin (amoxicillin and clavulanic acid), 875 mg twice daily for 7 days	Faecal; TRF analysis, PCR and enzyme digest, bacterial culturing on selective media for enumeration of different species	Analysis of faecal terminal restriction fragment length polymorphism data for treatment effects of probiotic treatment concurrent with antibiotic therapy	There was large subject to subject variability. Subjects fell into two categories, those with stable baseline microbiota and those where it varied significantly. Antibiotics had a significant effect on faecal microbiota across all subjects. Culture data also had large variation in counts. Increasing trends were visible in Bacteroides and enterics at day 21. There was no trend seen for Clostridium, Bifidobacterium, and Lactobacillus. The placebo group had a significant change from baseline at day 21 in numbers of bacteria.
**Lode et al. 2001**	Germany	volunteers	N = 12 adults	Not specified	Healthy	Amoxicillin/clavulanic acid 1000mg (750 : 125 mg respectively) daily	Faecal; culture on selective agar, colonies counted, isolated and identified to genus level	investigate the ecological effects of linezolid, compared with those of amoxicillin/clavulanic acid, on the intestinal human microflora	Amoxicillin/Clavulanic acid was associated with significant increase in numbers of enterococci and E. coli in the aerobic intestinal microflora during administration. Other aerobic bacteria were largely unaffected. Anaerobic during administration, numbers of bifidobacteria, lactobacilli and clostridia decreased significantly, numbers of anaerobic cocci and Bacteroides were not markedly altered. *C. difficile* were isolated from 3 of the volunteers. Some resistant enterobacteria such as *E.*

For peer review only - http://bmjopen.bmj.com/site/about/guidelines.xhtml

									coli, *Klebsiella* sp. and *Enterobacter* sp. were isolated. The microflora was normalized 35 days after the end of administration.
**Nitrofurantoin**									
**Stewardson et al. 2015**	Switzerland	Prospective cohort	N = 40 adults; 10 nitrofuratonin	Ambulatory care	Lower UTI	Nitrofuratonin (100mg twice daily for 5 days)	Faecal by 16S rRNA and sequence analysis; operational taxonomic unit (OTU)	compare the effects of ciprofloxacin and nitrofurantoin on the gut microbiota composition of non-hospitalised patients with UTIs compared to patients without antibiotic exposure and household contacts of patients receiving ciprofloxacin	Ciprofloxacin caused changes in a number of genus of gut bacteria. Substantial recovery after 4 weeks. Nitrofuratonin treatment correlated with a reduced relative proportion of *Clostridium* and an increased proportion of *Faecalibacterium*, therefore minimal perturbation of intestinal microbiota
**Vervoort et al. 2015**	Belgium and Poland	Prospective cohort	N = 13; 5 controls	ambulatory patients visiting GPs	uncomplicated UTI	Nitrofurantoin (100 mg three times	Faecal; 16S rDNA PCR, sequencing and analysis	the impact of nitrofurantoin treatment on the gastrointestinal	Nitrofurantoin treatment did not show any significant impact on the faecal microbiota other than a temporary increase in the Actinobacteria phylum, more

For peer review only

						daily for 3-15 days)		flora of patients with uncomplicated UTIs	specifically in the beneficial Bifidobacterium genus.
**Mavromanolakis et al. 1997**	Greece	randomised	N = 21 women; 7 with treatment	Not specified	UTI (at least 3 episodes caused by Enterobacteriaceae in the preceding 12 months)	Nitrofurantoin (100 mg daily for 30 days)	Faecal samples (and urine) cultured and plated on selective agar, API test kits for identification	impact of doses of norfloxacin, trimethoprim-sulfamethoxazole and nitrofurantoin on aerobic bowel flora	Before antibiotic treatment all stools contained Enterobacteriaceae and *Enterococcus* spp. Nitrofurantoin did not alter either the enterobacteria or enterococci of the colonic flora dung treatment. No resistant strains of gram negative aerobic bacteria were detected no patient receiving the drug was found to be colonised by yeasts. (NB. the other antibiotics tested did have an effect)
**Doxycycline**									
**Walker et al. 2005**	USA	RCT	N = 69 adult; 55 analysed	Clinic	Periodontitis	20 mg doxycycline	Faecal samples plated on a number of selective agar and incubated for CFU counts	To determine if a 9-month regimen of sub optimal doxycycline had an effect on either the intestinal or the vaginal microflora	The only statistically significant differences between the two treatment groups occurred in the doxycycline-resistant counts at the baseline sample. No between-treatment differences were detected at 3- or 9-month period either in the predominant bacterial taxa or in antibiotic susceptibilities
**Matto et al. 2008**	Finland	Not specified	N = 19; 10 controls	Not specified	Not specified	Doxycycline 150mg daily for 10 days	Faecal 16S rRNA PCR for Bifidobacteriu	To evaluate the influence of doxycycline	Bifidobacterium diversity was markedly higher in the control group than antibiotic group; each subject had 2-3 genotypes in the

						with probiotic	m sp and DGGE and culturing and sequencing	therapy on the composition and antibiotic susceptibility of intestinal bifidobacteria	control group; 0-3 in the antibiotic group. The isolated bifidobacteria represented *B. adolescentis/ ruminantium*, *B. longum*, *B. catenulatum/* pseudocatenulatum, *B. bifidum* and *B. dentium*. Tetracycline-resistant Bifidobacterium isolates were more commonly detected in the antibiotic group than in the control group, thus increasing the pool of resistant commensal bacteria in the intestine.
**Clarithromycin**									
**Brismar et al. 1991**	Sweden	Healthy volunteers	N = 10 adults	Not specified	Healthy	Clarithromyc in 250mg twice daily for 7 days	Culture on selective and non-selective agar	To compare the effect of clarithromycin and erythromycin on the normal flora	Clarithromycin decreased the numbers of streptococci and enterobacteria. Lactobailli, bifidobacterial and bacteroides were suppressed in the anaerobic microflora.
**Edlund et al. 2000**	Sweden	Healthy volunteers	N = 12 male adult	Not specified	Healthy	Clarithromyc in 500 mg twice daily for 7 days	Faecal; inhibition assay, culture on selective and non-selective agar	to investigate the ecological effects of moxifloxacin with those of clarithromycin on the intestinal human microflora	Clarithromycin significantly reduced *E. coli* in numbers during treatment but returned to pre-treatment levels at day 35. 6 subjects were colonised by resistant aerobic Gram negative rods, Citrobacter, Klebsiella, Proteus and Pseudomonas. No overgrowth of yeasts. Total

									number of anaerobic bacteria decreased during treatment. Bifidobacteria were eliminated or strongly reduced. Bacteroides declined. Lactobacilli and clostridia were markedly declined but not significantly. Only minor alterations in numbers of peptostreptococci, veillonellacocci. No overgrowth of *C. difficile*.
**Edlund et al. 2000**	Sweden	Randomised double blind controlled study	N = 20 adults	Not specified	Healthy	Clarithromyc in 500 mg for 10 days	Faecal; culture on selective agar aerobic and anaerobic, Gram stain and biochemical tests, gas liquid chromatograp hy	assess the impact of antibiotic on intestinal microflora	Moderate disturbances in aerobic intestinal microflora, number of *E. coli* significantly reduced at day 10. Overgrowth of *Klebsiella*, *Citrobacter* and *Enterobacter* spp. occurred in 6 subjects receiving clarithromycin. No significant overgrowth of Candida spp. There was a marked significant reduction of lactobacilli and bifidobacteria which persisted at day 24. Numbers of peptostroptococci, streptococci, clostridia, veillonella and *Bacteroides* spp and total anaerobic bacteria was not significantly affected.

**Phenoxymethylpenicillin**									
**Adamsson et al. 1997**	Sweden	Healthy volunteers	N = 20 adults; 10 with treatment	Not specified	healthy	Phenoxymethylpenicillin 1 g twice a day for 10 days	Faecal antimicrobial assay and culture	to investigate the ecological effects of phenoxymethylpenicillin, on the oropharyngeal and intestinal human microflora	Numbers of viridans streptococci, enterococci and Bacillus were unaffected. There were minor alterations in numbers of *E. coli*. Three subjects became colonised with Klebsiella and one with high numbers of non-fermentative gram-negative rod. Numbers of Clostridium increased. Bacteroides were unaffected. The mean numbers of total aerobic intestinal microorganisms did not alter significantly during phenoxymethylpenicillin administration. No significant changes were noticed in the total numbers of anaerobic intestinal bacteria.

Table 4. Impact of Amoxicillin on the gastrointestinal microflora.

Study	Dose mg/day	Days of administration	Number of patients	Impact on							Overgrowth	
				**Aerobic G+ cocci**	**Pseudomonas**	**Enterobacteria**	**Anaerobic**	**Lactobacillus**	**Bifidobacteria**	**Bacteriodes**	**Clostridium**	**Candida**
Christensson et al 1991	250x3	7	38	No change	isolated 4 patients	Increase		Increase	Increase	Increase	Isolated 2 patients	Isolated 10 patients
Floor et al 1994	500x3	7	40	Slight change	-	Increase	No change	-	-	-	-	Increase
Mangin et al 2010	50/kg/d	7	31	-	-	-	-	-	Changes in diversity	-	-	-
Monreal et al 2005	-	-	22	-	-	-	-	Decrease	Decrease	Decrease	-	-
Ladirat et al 2014	375x3	5	12	-	-	Overgrowth	-	-	Decrease	-	-	-
Brismar et al 1993	500x3	7	10	Minor decrease	-	Increase	No change	No change	No change	No change	None	No change
Edlund et al	500x3	7	10	-	-	Overgrowth	-	No change	No change	No change	No change	No change
De La Cochetiere et al 2005*	500x3	6	5	-	-	-	-	-	-	-	-	-
							**Fusobacteriaceae**	**Lachnospiraceae**	**Bifidobacteria/Erysipelotrichaceae**	**Bacteriodales/Veillonellaceae/Porphyromonadaceae**		
Abeles et al 2016†	500x2	3/6	6/6				Increase	Diminished even after 6 mo	Decrease then increase	Decrease		

For peer review only

Notes

- = not reported

* this paper measured similarity indices of TGGE profiles and showed shifts in dominant species upon antibiotic treatment

† also a reduction in diversity

For peer review only

Table 5. Impact of Amoxicillin with Clavulanic acid on the gastrointestinal microflora.

Study	Dose mg/day	Days of administration	Number of patients	Impact on							Overgrowth	
				**Aerobic G+ cocci**	**Pseudomonas**	**Enterobacteria inc. *E. coli***	**Anaerobic cocci**	**Lactobacillus**	**Bifidobacteria**	**Bacteriodes**	**Clostridium**	**Candida**
Lode et al	875/125	7	12	Increase	-	Increase	Little change	Decrease	Decrease	Little change	Decrease	
Young & Schmidt 2004*	875/125x2	10	1	-	-	Clusters present	-	-	Clusters present	Clusters present	Two clusters	
Engelbrektson et al 2009†	875/?x2	7	32	-	-	Increase	-	No trend	No trend	Increase	No trend	
Forssten et al 2014†	875/125	7	80			Increased		Not affected in probiotic group	Decreased in the placebo group		Decreased	
Korpela et al 2016**	-	-	142					Slight decrease		Two fold elevated Parabacteroides		

*this paper reported shifts in the representation of the major bacterial groups

†probiotic also given

** and penicillin V

For peer review only

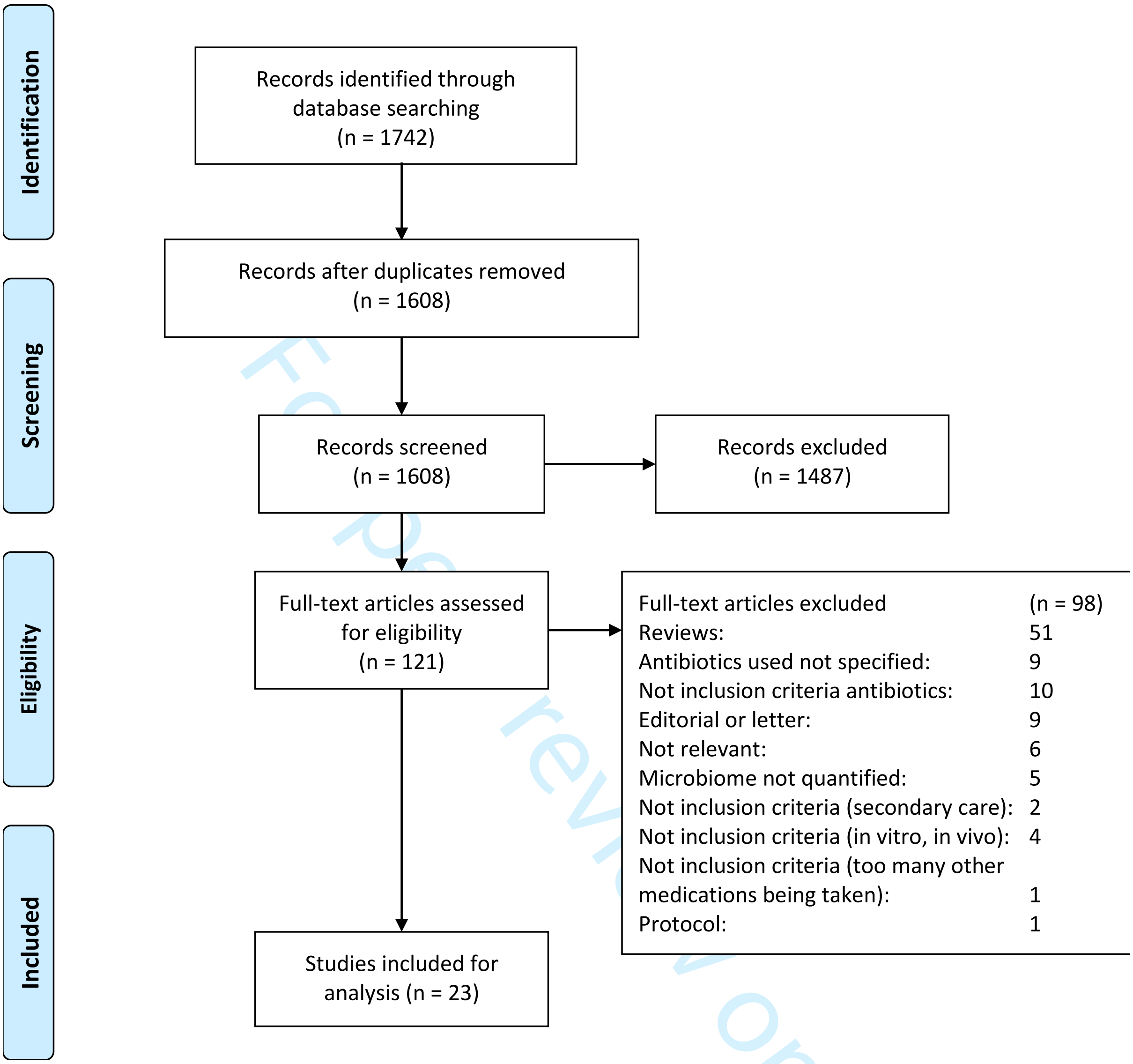

Figure 1. PRISMA flow chart for study selection.

Figure 2. Overall risk of bias assessment.

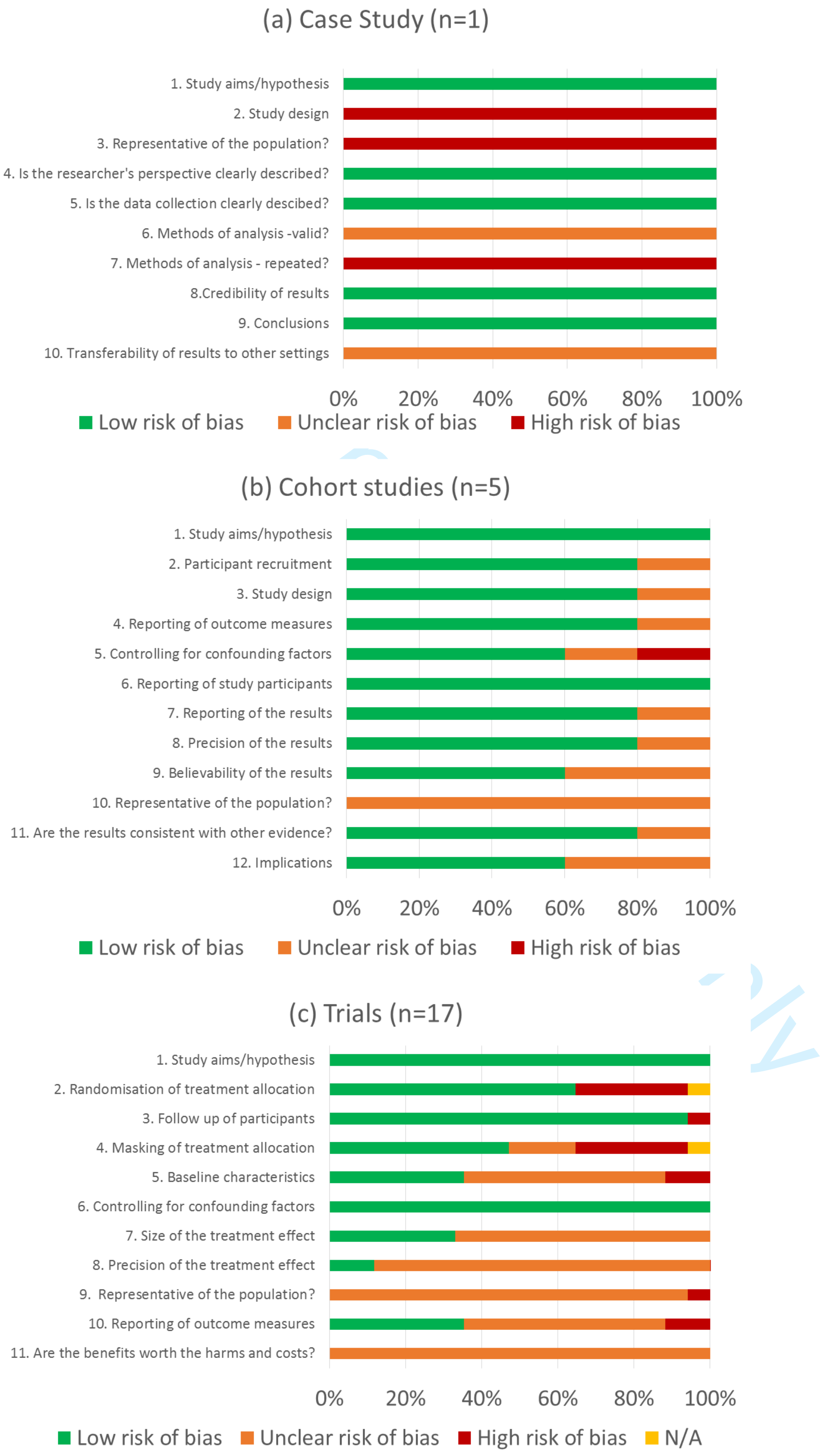

PRISMA 2009 Checklist

Section/topic	#	Checklist item	Reported on page #
**TITLE**			
Title	1	Identify the report as a systematic review, meta-analysis, or both.	1
**ABSTRACT**			
Structured summary	2	Provide a structured summary including, as applicable: background; objectives; data sources; study eligibility criteria, participants, and interventions; study appraisal and synthesis methods; results; limitations; conclusions and implications of key findings; systematic review registration number.	2-3
**INTRODUCTION**			
Rationale	3	Describe the rationale for the review in the context of what is already known.	4-6
Objectives	4	Provide an explicit statement of questions being addressed with reference to participants, interventions, comparisons, outcomes, and study design (PICOS).	6
**METHODS**			
Protocol and registration	5	Indicate if a review protocol exists, if and where it can be accessed (e.g., Web address), and, if available, provide registration information including registration number.	6
Eligibility criteria	6	Specify study characteristics (e.g., PICOS, length of follow-up) and report characteristics (e.g., years considered, language, publication status) used as criteria for eligibility, giving rationale.	7; Table 2
Information sources	7	Describe all information sources (e.g., databases with dates of coverage, contact with study authors to identify additional studies) in the search and date last searched.	6-7
Search	8	Present full electronic search strategy for at least one database, including any limits used, such that it could be repeated.	Table 1
Study selection	9	State the process for selecting studies (i.e., screening, eligibility, included in systematic review, and, if applicable, included in the meta-analysis).	7
Data collection process	10	Describe method of data extraction from reports (e.g., piloted forms, independently, in duplicate) and any processes for obtaining and confirming data from investigators.	8
Data items	11	List and define all variables for which data were sought (e.g., PICOS, funding sources) and any assumptions and simplifications made.	8
Risk of bias in individual studies	12	Describe methods used for assessing risk of bias of individual studies (including specification of whether this was done at the study or outcome level), and how this information is to be used in any data synthesis.	8-9
Summary measures	13	State the principal summary measures (e.g., risk ratio, difference in means).	8-9
Synthesis of results	14	Describe the methods of handling data and combining results of studies, if done, including measures of consistency (e.g., I^2) for each meta-analysis.	9

For peer review only

For peer review only - http://bmjopen.bmj.com/site/about/guidelines.xhtml

PRISMA 2009 Checklist

Section/topic	#	Checklist item	Reported on page #
Risk of bias across studies	15	Specify any assessment of risk of bias that may affect the cumulative evidence (e.g., publication bias, selective reporting within studies).	8-9
Additional analyses	16	Describe methods of additional analyses (e.g., sensitivity or subgroup analyses, meta-regression), if done, indicating which were pre-specified.	Not applicable
**RESULTS**			
Study selection	17	Give numbers of studies screened, assessed for eligibility, and included in the review, with reasons for exclusions at each stage, ideally with a flow diagram.	9; Figure 1
Study characteristics	18	For each study, present characteristics for which data were extracted (e.g., study size, PICOS, follow-up period) and provide the citations.	Table 3
Risk of bias within studies	19	Present data on risk of bias of each study and, if available, any outcome level assessment (see item 12).	10-12; Figure 2
Results of individual studies	20	For all outcomes considered (benefits or harms), present, for each study: (a) simple summary data for each intervention group (b) effect estimates and confidence intervals, ideally with a forest plot.	12-21
Synthesis of results	21	Present results of each meta-analysis done, including confidence intervals and measures of consistency.	Not applicable
Risk of bias across studies	22	Present results of any assessment of risk of bias across studies (see Item 15).	Not applicable
Additional analysis	23	Give results of additional analyses, if done (e.g., sensitivity or subgroup analyses, meta-regression [see Item 16]).	Not applicable
**DISCUSSION**			
Summary of evidence	24	Summarize the main findings including the strength of evidence for each main outcome; consider their relevance to key groups (e.g., healthcare providers, users, and policy makers).	21-24
Limitations	25	Discuss limitations at study and outcome level (e.g., risk of bias), and at review-level (e.g., incomplete retrieval of identified research, reporting bias).	22-23
Conclusions	26	Provide a general interpretation of the results in the context of other evidence, and implications for future research.	23-24
**FUNDING**			
Funding	27	Describe sources of funding for the systematic review and other support (e.g., supply of data); role of funders for the systematic review.	24

From: Moher D, Liberati A, Tetzlaff J, Altman DG, The PRISMA Group (2009). Preferred Reporting Items for Systematic Reviews and Meta-Analyses: The PRISMA Statement. PLoS Med 6(7): e1000097. doi:10.1371/journal.pmed1000097

For peer review only - http://bmjopen.bmj.com/site/about/guidelines.xhtml

PRISMA 2009 Checklist

For more information, visit: www.prisma-statement.org.

For peer review only

BMJ Open

Antibiotic-induced changes in the human gut microbiota for the most commonly prescribed antibiotics in primary care in the United Kingdom: systematic review

Journal:	*BMJ Open*
Manuscript ID	bmjopen-2019-035677.R1
Article Type:	Original research
Date Submitted by the Author:	12-Jun-2020
Complete List of Authors:	Elvers, Karen; University of Bristol, Bristol Medical School<br>Wilson, Victoria; University of Bristol, Bristol Medical School<br>Hammond, Ashley; University of Bristol, Bristol Medical School<br>Duncan, Lorna; University of Bristol, Bristol Medical School<br>Huntley, Alyson; University of Bristol, Bristol Medical School<br>Hay, Alastair; University of Bristol, Bristol Medical School<br>van der Werf, Esther; University of Bristol Medical School; Louis Bolk Institute
**Primary Subject Heading**:	General practice / Family practice
Secondary Subject Heading:	Evidence based practice
Keywords:	PRIMARY CARE, Urinary tract infections < UROLOGY, Respiratory infections < THORACIC MEDICINE, Microbiology < PATHOLOGY

SCHOLARONE™
Manuscripts

BMJ

I, the Submitting Author has the right to grant and does grant on behalf of all authors of the Work (as defined in the below author licence), an exclusive licence and/or a non-exclusive licence for contributions from authors who are: i) UK Crown employees; ii) where BMJ has agreed a CC-BY licence shall apply, and/or iii) in accordance with the terms applicable for US Federal Government officers or employees acting as part of their official duties; on a worldwide, perpetual, irrevocable, royalty-free basis to BMJ Publishing Group Ltd ("BMJ") its licensees and where the relevant Journal is co-owned by BMJ to the co-owners of the Journal, to publish the Work in this journal and any other BMJ products and to exploit all rights, as set out in our licence.

The Submitting Author accepts and understands that any supply made under these terms is made by BMJ to the Submitting Author unless you are acting as an employee on behalf of your employer or a postgraduate student of an affiliated institution which is paying any applicable article publishing charge ("APC") for Open Access articles. Where the Submitting Author wishes to make the Work available on an Open Access basis (and intends to pay the relevant APC), the terms of reuse of such Open Access shall be governed by a Creative Commons licence – details of these licences and which Creative Commons licence will apply to this Work are set out in our licence referred to above.

Other than as permitted in any relevant BMJ Author's Self Archiving Policies, I confirm this Work has not been accepted for publication elsewhere, is not being considered for publication elsewhere and does not duplicate material already published. I confirm all authors consent to publication of this Work and authorise the granting of this licence.

For peer review only

Antibiotic-induced changes in the human gut microbiota for the most commonly prescribed antibiotics in primary care in the United Kingdom: systematic review

Karen T Elvers, senior research associate[2], Victoria J Wilson, senior research associate (0000-0002-1657-3861)[2], Ashley Hammond, senior research associate[2], Lorna Duncan, senior research associate[2], Alyson L Huntley, senior research fellow (0000-0001-9409-7891) [2], Alastair D Hay, professor of primary care (0000-0003-3012-375X)[2], Esther T van der Werf, senior lecturer in epidemiology (0000-0002-5330-5735)[1, 2]

[1]Louis Bolk Instituut, Kosterijland 3-5, 3981 AJ, Bunnik, The Netherlands

[2]Centre for Academic Primary Care, Population Health Sciences, Bristol Medical School, University of Bristol, Canynge Hall, 39 Whatley Road, Bristol, BS8 2PS

Corresponding author: Esther T. van der Werf, Tel: +31 6 86877696

e.vanderwerf@louisbolk.nl

Karen T Elvers karen.elvers@bristol.ac.uk

For peer review only - http://bmjopen.bmj.com/site/about/guidelines.xhtml

19 Victoria J Wilson victoria.wilson@bristol.ac.uk

20 Ashley Hammond ashley.hammond@bristol.ac.uk

21 Lorna Duncan lorna.duncan@bristol.ac.uk

22 Alyson Huntley alyson.huntley@bristol.ac.uk

23 Alastair D Hay alastair.hay@bristol.ac.uk

24

25 Short running title: The human gut microbiota and commonly prescribed antibiotics

For peer review only

Abstract

Objective The gut microbiota influences many aspects of human health. We investigated the magnitude and duration of changes in gut microbiota in response to antibiotics commonly prescribed in UK primary care.

Methods We searched MEDLINE, EMBASE and AMED to May 2020 including all study designs, collecting and analysing data on the effect of antibiotics prescribed for respiratory and urinary tract infections. We followed PRISMA and Cochrane standard methods. Risk of bias was evaluated using the Critical Appraisal Skills Programme. Narrative synthesis was used to report the themes emerging from the data.

Main outcome measures Primary outcomes were antibiotic-induced changes in the composition and/or diversity of the gut microbiota. Secondary outcome was the time for the microbiota to return to baseline.

Results Thirty one articles with low or unclear risk of bias showed antibiotics impact the gut microbiota by causing rapid and diminished levels of bacterial diversity and changes in relative abundances. After cessation of treatment, gut bacteria recover, in most individuals, to their baseline state within a few weeks. S,ome studies suggested longer term effects from two to six months. Considerable heterogeneity in methodology makes the studies prone to biases and other confounding factors. Doxycycline was associated with a marked short-term decrease in *Bifidobacterium* diversity. Clarithromycin decreased the populations of Enterobacteria, and the anaerobic bacteria *Bifidobacterium* sp. and *Lactobacillus* sp. in numbers and diversity for up to 5 weeks. Phenoxymethylpenicillin, nitrofurantoin and amoxicillin had very little effect on the gut microbiome.

For peer review only - http://bmjopen.bmj.com/site/about/guidelines.xhtml

Conclusions Despite substantial heterogeneity of the studies and small sample sizes, there is evidence that antibiotics commonly used in primary care influence the composition of the gastrointestinal microbiota. Larger population-based studies are needed to fully understand how antibiotics modulate the microbiota, and to determine if these are associated with (longer term) health consequences. Review registration number CRD42017073750 http://www.crd.york.ac.uk/PROSPERO

For peer review only

For peer review only - http://bmjopen.bmj.com/site/about/guidelines.xhtml

Strengths and limitations of this study

- This review addresses the antibiotic-induced changes in gut microbiota for the most commonly prescribed antibiotics in UK primary care for the infections most frequently seen in general practice: respiratory tractand urinary tract infections.
- The study used a complete and inclusive search strategy complemented by manually scanning reference lists of identified articles for further relevant publications.
- We searched three databases, all years up to May 2020, for any study type, with clearly defined criteria for study inclusion.
- It was not feasible to combine results in a meta-analysis as the majority of the studies were small, poorly randomised and with limited follow up. There was considerable heterogeneity in methodology which makes them prone to biases and other confounding factors.
- This limits our understanding of the long term changes induced by commonly prescribed antibiotics, the ability to modify antibiotic treatment to each situation and to make recommendations to clinicians.

For peer review only - http://bmjopen.bmj.com/site/about/guidelines.xhtml

Introduction

In the UK, between 2000 and 2014, antibiotic prescriptions increased from 14.3 to 19.7 defined daily doses (DDDs) per 1,000 inhabitants per day. The volume of antibiotic prescribing has since decreased, reaching 18.7 DDDs per 1,000 inhabitants per day in 2016 (http://www.oecd.org/). Misuse and overuse of antimicrobials, not only in medical sectors, but also veterinary and agricultural, results in increasing resistance which globally limits our ability to control infections. Decreasing antibiotic consumption is a key target to dealing with the problem and the UK has implemented antimicrobial stewardship interventions in primary care[1] and detailed a five year action plan for tackling antimicrobial resistance.[2]

Data analysed from The Health Improvement Network (THIN) database, stated a median prescribing rate among participating practices was 626 antibiotic prescriptions/1000 patients (IQR 543-699), 69% of which could be linked to a body system and/or clinical condition.[3] Of these prescriptions, 46% were linked to respiratory tract infections and 22.7% to infections of the urogenital tract. About half of all antibiotics prescribed were penicillin, of which approximately 55% was amoxicillin, this is followed by 13% macrolide use and 12% tetracycline.[3] General practice (GP) consultation rates in England and Wales show that a quarter of the population will visit their GP because of a respiratory tract infection (RTI) each year.[4] Antibiotic use to treat such infection varies between individual clinicians and countries,[4-6] but accounts for 60% of all antibiotic prescribing in general practice.[7-9] Similarly, urinary tract infections (UTI) are commonly seen in primary care.[10] Patients are frequently treated with antibiotics, even though the infection often is not microbiologically confirmed.[11] The overuse of antibiotics for conditions that are not serious or where antibiotic treatment is

For peer review only

For peer review only - http://bmjopen.bmj.com/site/about/guidelines.xhtml

not appropriate for the illness, has led to their reduced effectiveness and emergence of resistant bacteria.

The normal gut microbiota consists of approximately 800–1000 different bacterial species and greater than 7000 different strains.[12] ‘Normal’ is defined as the commensal species predominantly and consistently found in the gut of healthy human. The composition of the oesophageal and gastric microbiomes is distinct from that of the colon. There is a greater diversity of species and number per gram of contents in the colon, reflecting its higher pH.[13 14] The core microbiome in healthy individuals is dominated by phyla Firmicutes and Bacteroidetes (over 90%), followed by Verrucomicrobia and Actinobacteria.[13] Some essential functions of a healthy gut microbiota include vitamin production, nutrient metabolism, immunomodulation and protection against infection by inhibiting colonisation of the gut by pathogens. Studies into gut microbiomes from different countries have identified differences in diversity which reflect the differences in diet, geography, early-life exposures and genetics.[15] Age, diet, antibiotics, probiotics and prebiotics all contribute to the shaping of the healthy gut microbiota and this continues dynamically throughout life.[13 14 16]

These differences lead to microbiome dysbiosis, with increasing evidence that alterations to the human microbiome can affect how well the immune system functions and its ability to resist infection,[17 18] increase the risk of developing Parkinson’s disease, multiple sclerosis and Alzheimer’s disease,[19] the risk of depression, anxiety, or psychosis[20] and indirectly affect the health long term and risk of obesity and diabetes.[21] There is increasing evidence that the microbiome is crucial in a cancer patient's risk of infectious complications.[22] Recent studies have shown that antibiotic exposure in childhood is associated with increased risk for several

For peer review only - http://bmjopen.bmj.com/site/about/guidelines.xhtml

diseases including obesity, types 1 and 2 diabetes, inflammatory bowel diseases, Crohn's disease, coeliac disease, autoimmune diseases such as allergies, juvenile idiopathic arthritis and asthma.[23-26] It appears that these effects are most pronounced if the antibiotics are consumed within the first two years of life and the effects may well be cumulative.[17 27 28] This is particularly important in young children, when the adult microbiome has not been fully established.[29 30] Once antibiotic treatment has stopped, the microbiome may present a certain degree of resilience, being capable of returning to a composition similar to the original one, but this is poorly understood and can take months or even years.[17 31]

Differences in perceptions about how risk free antibiotic treatment is, may in part account for the enormous variation in rates of their use from practitioner to practitioner, between localities and across countries, but also within a specific group of practitioners such as GPs. The real cost of the most commonly prescribed antibiotics in primary care must be clearly understood, including the differences between particular antimicrobial agents in their effects on the microbiome. This information will be specifically useful for antimicrobial stewardship in primary care.

Aim

To investigate the antibiotic-induced changes in gut microbiota for the most commonly prescribed antibiotics in primary care in the UK: those prescribed for respiratory tract infections and urinary tract infections. Our primary outcome was defining any antibiotic-induced changes in the composition of the gut microbiome, measured by 1) abundance and/or 2) diversity of the microbes, whilst our secondary outcome measured the time that is needed to restore the microbiome to a pre-antibiotic state.

For peer review only

For peer review only - http://bmjopen.bmj.com/site/about/guidelines.xhtml

Methods

A systematic literature review of studies that evaluated association between antibiotics commonly prescribed for respiratory tract and urinary tract infections in primary care and their effect on the gut microbiome was conducted. Preferred Reporting Items for Systematic Reviews and Meta-analyses (PRISMA) guidelines and checklist were used as a framework for this review (Table S1 PRISMA checklist).[32] The study protocol was registered with PROSPERO number, CRD42017073750 http://www.crd.york.ac.uk/PROSPERO.

Search Strategy

Electronic databases MEDLINE, EMBASE and AMED, were used to identify relevant articles up to May 2020, in English and available as full text. A combination of search terms included the following keywords: (antibacterial agents OR bacterial infections) AND (microbiota OR microbiome). The strategy was designed to identify studies conducted in any country, investigating antibiotic-induced changes in the human gut microbiota; analysed at either the individual or population-level, published in any language (Table 1). Reference lists of included studies and relevant reviews were hand searched to identify additional eligible studies. All selected abstracts and citations were exported from the scientific databases to the reference management software EndNote X9 (Thompson Reuters, New York, NY, USA) and duplicates excluded.

Table 1. Search strategy

1. Anti-Bacterial Agents/ae, pd, tu
2. Bacterial Infections/dt, mi

For peer review only - http://bmjopen.bmj.com/site/about/guidelines.xhtml

3. Microbiota/<br>4. (Microbiome or Microbiomes or Gut microbiome or Gut microbiomes or Microbiota Gut or microbiota).mp.<br>5. Humans/<br>6. 1 or 2<br>7. 3 or 4<br>8. 6 and 7<br>9. 5 and 8

159

160 **Study selection and eligibility criteria**

161 A study was eligible for review if it reported quantitative changes to the gut microbiota due
162 to treatment with antibiotics most commonly prescribed for respiratory and urinary tract
163 infections prescribed in primary care (Table 2). Respiratory tract infections included both
164 upper respiratory tract infections (URTIs), including the common cold, laryngitis,
165 pharyngitis/tonsillitis, acute rhinitis, acute rhinosinusitis and acute otitis media and lower
166 respiratory tract infections (LRTIs), including acute bronchitis, bronchiolitis, pneumonia and
167 tracheitis. We included uncomplicated and recurrent urinary tract infections (UTI and rUTIs).
168 We excluded any animal studies or hospital-based studies.

169 **Table 2. PICOS criteria for inclusion and exclusion of studies.**

**Parameter**	**Inclusion**	**Exclusion**
Participants	Humans adults and children	Animals, *in vitro*

For peer review only

For peer review only - http://bmjopen.bmj.com/site/about/guidelines.xhtml

Intervention	Specific antibiotics of interest prescribed in primary care	Other antibiotics
Comparator	Negative controls or other antibiotics	None
Outcomes	Changes in gut microbiota, total numbers, diversity and composition; time taken to recover	Antimicrobial resistance
Study design	All study types	None

The main exposure of interest in this review was the effect commonly prescribed antibiotics have on the gut microbiome based on prescriptions advice included in the NICE guidelines [1 8 33-35] and on current use as described in nation reports on antibiotic use.[1 34] Of particular interest are those antibiotics, which are commonly used in primary care for the treatment of both upper and lower RTIs (amoxicillin, phenoxymethylpenicillin, doxycycline, co-amoxiclav, erythromycin, clarithromycin) and UTIs (trimethoprim, nitrofurantoin, amoxicillin, cephalexin).

Screening, data extraction and management

Five reviewers (VW, KE, EvdW, LD and AH) independently screened search results and assessed each potential study according to the inclusion and exclusion criteria. All decisions were recorded on a spreadsheet. Full text papers for all eligible studies were obtained and three reviewers (VW, KE, AH) independently screened the selected papers a second time.

For peer review only - http://bmjopen.bmj.com/site/about/guidelines.xhtml

The following data were extracted from the included studies into an Excel spreadsheet by one reviewer (KE) and a percentage verified for accuracy by another (VW): author, journal, year of publication, study design, study country, number of participants, number of participants of interest to this review, age range, percentage of female participants, other inclusion criteria, exclusion criteria, recruitment methods, recruitment time period, sample type, primary outcome, secondary outcome, antibiotic (of interest to this review) used, any other antibiotics used, dose of antibiotics (of antibiotic of interest to this review), time course (of antibiotic of interest to this review), number of timepoints for collecting samples, time points for each sample collection, testing method, infection or condition, overall conclusion. A tabular summary was developed for this review, which included study ID, country, design, population, setting, infection, antibiotic of interest, sample and analysis, primary outcome and main results (Table 3).

For peer review only - http://bmjopen.bmj.com/site/about/guidelines.xhtml

Table 3. Summary of studies included in the review

Study ID	Country	Design	Population	Setting	Infection	Antibiotic of interest (and dosage)	Sample and analysis	Primary Outcome	Results
<u>**Amoxicillin**</u>									
**De La Cochetiere et al. 2005**[36]	France	Cohort	N = 6 adults	Volunteers	Healthy	Amoxicillin (500mg three times a day for 5 days)	Faecal samples; 16S rRNA PCR and TTGE gel analysis and sequence analysis	To assess the ability of the human faecal microbiota to return to its original dominant species profile after a 5-day course of amoxicillin	Similarity indices of the TTGE profiles on D1 ranged from 93-99%; on D3 62-82% and D4 46-94%. On D30 and D60 these had raised back to 65-95% and 66-98% respectively. Bands excised from the gel related to *Clostridium nexile* and *Ruminococcus torques* (and β-Proteobacteria). No normalisation of bacterial abundance d55 after antibiotic treatment.
**Monreal et al. 2005**[37]	Brazil	Prospective	N = 42 adults; 20 controls	Emergency departmen t; blood donor centres	Bacterial respiratory infections (sinusitis, pneumonia)	Amoxicillin	Faecal samples analysed by culture and CFU on blood agar	Investigated the influence of respiratory tract infections and of amoxicillin therapy on the normal intestinal	Concentration of *Bacteriodes*, *Bifidobacterium* spp. *Lactobacillus* spp. were significantly decreased in the antibiotic group. After 30 days abundance of Bifidobacterium and Lactobacillus had normalised.

For peer review only

For peer review only - http://bmjopen.bmj.com/site/about/guidelines.xhtml

								microbiota of patients	
**Pallav et al. 2014**[38]	USA	RCT	N = 24; 8 with amoxicillin, 8 controls, 8 other	Clinical and translation al Science Centre	Healthy	Amoxicillin 250mg three times a day for 7 days	16S rRNA gene sequencing region ns Roche 454 GS FLX Titanium GreenGenes/ RDP/NCBI Database	Effect of amoxicillin versus controls	The genera which are most abundant are Faecalibacterium, Bacteroides, Rosburia, Clostridium, and Ruminococcus. No change in bacterial abundance in control group. The most notable change was an increase in Escherichia/Shigella during antibiotic treatment. Also increase in Bacteriodes but decrease in *Faecalibacterium* spp. Antibiotic associated changes persisted to the end of the study, 42 days after antibiotic therapy ended.
**Abeles et al. 2016**[39]	USA	Cohort of unrelated cohabiting individuals randomly assigned	N = 56 adults	University campus	Self reported health status	Amoxicillin 500 mg twice daily for 3 and 7 days	16S rRNA amplified from DNA using QIAGEN stool Mini Kit; sequencing	Effects of commonly prescribed antibiotics on microbiota	Microbiota grew more dissimilar over time but not significant. Most abundant taxa in gut were Bacteroidaceae, Lachnospiraceae and Ruminococcaceae. Lachnospiraceae, Veillonellaceae, Bacteroidales and Porphyromonadaceae were significantly decreased. Fusobacteriaceae increased. Bifidobacteriales and Erysipelotrichaecae initially decreased. There were sustained reductionsin microbiome diversity in response to amoxicillinover 6 months.

For peer review only - http://bmjopen.bmj.com/site/about/guidelines.xhtml

**Mangin et al. 2010[40]**	Chile	Study trial	N = 42 infants; 31 with treatment	Health centre	Acute bronchitis	Amoxicillin 50 mg/kg/d in three daily doses for 7 days	Faecal; real time PCR for Bifidobacterium, TTGE and sequence analysis	to investigate the quantitative and qualitative changes occurring in the faecal bifidobacterial populations in 18 month children after a one-week amoxicillin treatment	Total bacteria and numbers of Bifidobacteria were not significantly altered by amoxicillin treatment. However, treatment changed species diversity, a complete disappearance of *B. adolescentis*, increase in *B. bifidum*. No effect on *B. longum*, *B. pseudocatenulatum*/*B. catenulatum*.
**Christensson et al. 1991[41]**	Sweden	Randomised double blind parallel multicentre trial	N = 84 adults; 44 with treatment (38 /44 completed)	outpatients	Lower respiratory tract infection	Amoxicillin 250 mg twice daily for 7 days	Faecal; cultured aerobically and anaerobically in broth to ID pathogenic microorganism and on selective plates for	To compare cefaclor and amoxycillin as treatment for lower respiratory tract infections and in their ability to influence colonization resistance	No change in abundance of *Enterococcus* spp., *Staphylococcus* spp. or *Streptococcus* spp. while enterobacteria significantly increased. *Pseudomonas* sp. and *Candida albicans* were isolated from some samples. Anaerobic cocci numbers were reduced in some patients and increased in others. Lactobacilli, bifidobacteria, bacteriodes and eubacteria increased. A couple of patients were colonised with *C. difficile*.

For peer review only - http://bmjopen.bmj.com/site/about/guidelines.xhtml

							analysis of microflora changes		
**Brismar et al. 1993**[42]	Sweden	single blind randomised trial	N = 20 adults; 10 with treatment	Not specified	Healthy volunteers	Amoxicillin 500 mg every 8 h for 7 days	Faecal; selective media, MICs, beta lactamase activity	to study the oral and intestinal microflora before, during and after administration of cefpodoxime proxetil and amoxicillin	Numbers of aerobic intestinal microflora were slightly affected by amoxicillin administration. A minor decrease in numbers of streptococci and staphylococci, an over growth of Klebsiella and Enterobacter in a couple of samples. No overgrowth of enterococci or yeasts occurred. Eubacteria was reduced by amoxicillin. Increase in amoxicillin-resistant Enterobactericaeae (*E. coli*, *Klebsiella* sp. and *Enterobacter* sp.) No change in abundance of *Bacteroides* spp., *Bifidobacterium* spp., *Enterococcus* spp., *Lactobacillus* spp. or yeast. No new colonisation with *C. difficile*. Normalisation of bacterial abundance 14 days after antibiotic treatment.
**Edlund et al. 1994**[43]	Sweden	Cohort	N = 44 adults, 10 with treatment	Not specified	Not specified	Amoxicillin 500 mg t.i.d for 7 days	Faecal; serial dilutions and selective plating aerobic and anaerobic	The investigation was focused on drug concentrations in faeces, beta lactamase	Administration of amoxicillin affected the aerobic intestinal microflora to a minor extent. There was an overgrowth of *Klebsiella* spp. and *Enterobacter* spp in some samples. No change in abundance of anaerobic bacteria, *Bacteriodes* spp., *Bifidobacterium* spp.

For peer review only - http://bmjopen.bmj.com/site/about/guidelines.xhtml

								production by the intestinal microflora, alterations in the microflora and susceptibility patterns	*Enterococcus* spp., *E. coli*, *Clostridium* spp., *Lactobacillus* spp. or yeast. No colonisation with *C. difficile*. Intestinal microflora returned to normal 2 weeks after treatment stopped.
**Ladirat et al. 2014[44]**	Netherlands	double blinded randomised parallel intervention study	N = 12 adults (2 not analysed due to non compliance)	Not specified	Healthy volunteers	Amoxicillin 375mg three times daily for 5 days	Faecal; Intestinal (I)-Chip microarray, total bacteria and Bifidobacterium spp using qPCR	determined the effects of prebiotic intake on the microbiota of healthy adult subjects during and after treatment with amoxicillin	Total bacteria and Bifidobacterium were similar in both groups before treatment. Numbers of Bifidobacterium decreased over time due to amoxicillin treatment in both groups, but more so in the antibiotic only group (no prebiotic). After antibiotic treatment numbers in the antibiotic only group were still lower than with prebiotics. Abundance of *Bifidobacterium* spp. *B. longum* and *B. thermophilum* was significantly higher in the prebiotic group. The composition of the microbiota in most subjects returned to normal after 3 weeks. Due to amoxicillin treatment, a decrease in the abundance of *Bifidobacterium* spp., an overgrowth of Enterobacteriaceae

For peer review only

**Floor et al. 1994[45]**	Netherlands	randomised double blind study	N = 80 adults	recruited from 9 General Practices	chronic bronchitis with purulent sputum	Amoxicillin, 500 mg 3 times a day for 7 days	Faecal; cultured aerobically and anaerobically on selective media, testing for *C. difficile*, enterococci, staphylococci, yeasts and biochemical identification	effect of both amoxicillin and loracarbef on oropharyngeal and intestinal microflora	No change in abundance of anaerobic bacteria or *E. faecalis*. Total numbers of aerobic Gram negative bacteria significantly higher on days 8-10 than at baseline. Enterococci cultured in 1 patient in amoxicillin group. Yeast numbers increased significantly but returned to baseline on days 21-28. Presence of other aerobic bacteria occurred in a slightly higher percentage of patients in the amoxicillin group. 13/15 new acquired bacteria in the amoxicillin group were Klebsiella. No new colonisation with *C. difficile*. Normalisation of bacterial and fungal abundance 21 days after treatment.
**Amoxicillin and Clavulanic acid**									
**Korpela et al. 2016[31]**	Finland	Part of a cohort from larger probiotic trial	N = 236 children; 142 donated faecal samples	Day care centres	Antibiotic use for respiratory (88%) other mainly urinary (5%)	penicillins (amoxicillin with or without clavulanic acid and penicillin V), macrolides (azithromyci	Faecal by 16S rRNA and sequence analysis; operational taxonomic unit (OTU); culture based antibiotic	Use of phylogenetics, metagenomics and antibiotic use on microbiota composition and metabolism	Macrolide use reduced abundance of Actinobacteria and increased Bacteriodetes and Proteobacteria. Penicillin groups did not have distinctly different phyla. Firmicutes was reduced. The effect of macrolide use was long lasting and associated with increased risk of asthma and antibiotic-associated weight gain.

						n and clarithromycin) and sulphonamide–trimethoprim	sensitivity testing		
**Mangin et al. 2012[46]**	France	Trial	N = 18 treated, no controls	Study centre	Healthy volunteers	875/125 mg oral dose of amoxicillin/clavulanic acid twice a day for 5 days	Faecal samples, Real-time PCR TTGE	amoxicillin-clavulanic acid treatment on total bacteria and on Bifidobacterium species balance in human colonic microbiota	Total bacterial concentrations as well as bifidobacteria concentrations were significantly reduced after antibiotic treatment. The mean similarity percentages of TTGE bacteria and TTGE bifidobacteria profiles were significantly reduced. Occurrence of *B. adolescentis*, *B. bifidum* and *B. pseudocatenulatum*/*B. catenulatum* species significantly decreased. Occurrence of *B. longum* remained stable. Moreover, the number of distinct Bifidobacterium species per sample significantly decreased. Bacterial abundance was not normalised 2 months after antibiotics stopped.
**Forssten et al. 2014[47]**	Finland	Randomised double blind	N = 80 adults; 40:40	Volunteers	Healthy	Augmentin (875mg Amoxicillin	Faecal samples; qPCR for	To investigate the effect of a specific	Generally Lactobacillus and Bifidobacterium were increased/restored in the probiotic group and reduced in the placebo group. Antibiotics

For peer review only - http://bmjopen.bmj.com/site/about/guidelines.xhtml

		placebo controlled parallel study	probiotic:placebo			and 125 mg clavulanate) for 7 days	specific microbial groups	combination of probiotic strains on the incidence of antibiotic induced microbiota disturbances	reduced levels of Clostridium in both groups, but increased Enterobacteriaceae
**Young et al. 2004[48]**	USA	Case report	N = 1 adult male	Not detailed	Acute sinusitis	amoxicillin-clavulanic acid (875 and 125 mg, respectively, twice daily for 10 days)	Faecal sample 16S rRNA PCR and sequencing and analysis	Molecular phylogenetic survey of the faecal microbiota from a patient who developed antibiotic associated diarrhoea during the administration of a broad-spectrum antibiotic	D0- sequences clustered within 4 bacterial groups; *Bacteroides* spp., *Clostridium* sp IV, *Clostridium* sp XIVa and *Bifidobacterium*. At D4- *Bacteroides* still major component but *B. distasonis* group not *B. fragilis* cluster. No *Clostridium* sp. XIVa or *Bifidobacterium* spp. 34% were now *Enterobacteriaceae*. D24- partial reversal, *B. fragilis* predominated and cluster XIVa returned, no *Enterobacteriaceae*, no *Bifidobacterium* spp. normalisation of abundance 14 days after treatment.

For peer review only

**Engelbreks ton et al. 2009[49]**	USA	probiotic-antibiotic study arm	N = 40 adults	Not specified	Healthy volunteers	Agumentin (amoxicillin and clavulanic acid), 875 mg twice daily for 7 days	Faecal; TRF analysis, PCR and enzyme digest, bacterial culturing on selective media for enumeration of different species	Analysis of faecal terminal restriction fragment length polymorphism data for treatment effects of probiotic treatment concurrent with antibiotic therapy	There was large subject to subject variability. Subjects fell into two categories, those with stable baseline microbiota and those where it varied significantly. Antibiotics had a significant effect on faecal microbiota across all subjects. Culture data also had large variation in counts. Increasing trends were visible in Bacteroides and enterics at day 21. There was no trend seen for Clostridium, Bifidobacterium, and Lactobacillus. The placebo group had a significant change from baseline at day 21 in numbers of bacteria.
**Lode et al. 2001[50]**	Germany	volunteers	N = 12 adults	Not specified	Healthy	Amoxicillin/clavulanic acid 1000mg (750 : 125 mg respectively) daily	Faecal; culture on selective agar, colonies counted, isolated and identified to genus level	investigate the ecological effects of linezolid, compared with those of amoxicillin/clavulanic acid, on the intestinal human microflora	Amoxicillin/Clavulanic acid was associated with significant increase in numbers of enterococci and *E. coli*. Other aerobic bacteria were largely unaffected. Anaerobic *Bifidobacterium* spp., *Clostridium* spp., *Lactobacillus* spp. decreased significantly. No change in abundance of *Bacteroides* spp., Gram positive bacilli or *Klebsiella* spp. *C. difficile* were isolated from 3 of the volunteers. Some resistant enterobacteria such as *E. coli*, *Klebsiella* sp. and *Enterobacter* sp. were isolated.

For peer review only - http://bmjopen.bmj.com/site/about/guidelines.xhtml

									The microflora was normalized 35 days after the end of administration.
**Kabbani et al 2017[51]**	USA	single-centre, open-label, randomized controlled	N = 49, 12 antiobiotic treated, 12 controls	Clinical and Translational Science Centre	Healthy volunteers	Amoxicillin/ clavulanate 875/125mg twice daily for 7 days	16S rRNA gene sequencing	To compare and contrast the effects of a probiotic and antibiotic, the main endpoint was change from baseline in the composition of the gut microbiota	Lower diversity (OTUs, Chao index) d10 and d21. Increased abundance of *Parabacteroides* spp. persisted 14d after antibiotic treatment was stopped. Control subjects had a stable microbiota throughout the study period. Significant microbiota changes in antibiotic treatment group included reduced prevalence of the genus Roseburia and increases in Escherichia, Parabacteroides, and Enterobacter. Microbiota alterations reverted toward baseline, but were not yet completely restored 2 weeks after treatment stopped.
**Nitrofurantoin**									
**Stewardson et al. 2015[52]**	Switzerland	Prospective cohort	N = 40 adults; 10 nitrofuratonin	Ambulatory care	Lower UTI	Nitrofuratonin (100mg twice daily for 5 days)	Faecal by 16S rRNA and sequence analysis; operational taxonomic unit (OTU)	compare the effects of ciprofloxacin and nitrofurantoin on the gut microbiota composition of non-hospitalised patients with	Ciprofloxacin caused changes in a number of genus of gut bacteria. Substantial recovery after 4 weeks. Nitrofuratonin treatment correlated with a non significant increase in *Clostridium* spp. and decrease in *Faecalibacterium* spp. No change in abundance of *Alistipes* spp., *Bacteroides* spp., *Bifidobacterium* spp., *Blautia* spp., *Dialister* spp., *Eubacterium* spp.

For peer review only - http://bmjopen.bmj.com/site/about/guidelines.xhtml

								UTIs compared to patients without antibiotic exposure and household contacts of patients receiving ciprofloxacin	*Oscillospira* spp., *Roseburia* spp. or *Ruminococcus* spp.
**Vervoort et al. 2015[53]**	Belgium and Poland	Prospective cohort	N = 13; 5 controls	ambulatory patients visiting GPs	uncomplicated UTI	Nitrofurantoin (100 mg three times daily for 3-15 days)	Faecal; 16S rDNA PCR, sequencing and analysis	the impact of nitrofurantoin treatment on the gastrointestinal flora of patients with uncomplicated UTIs	Nitrofurantoin treatment did not significantly impact on the faecal microbiota other than a temporary increase in the Actinobacteria phylum (*Bifidobacterium* spp.). No change in abundance of Bacteroidetes, Firmicutes, Proteobacteria, Tenericutes or Verrucomicrobia. Normalisation in abundance of Actinobacteria d31-43 after treatment.
**Mavromanolakis et al. 1997[54]**	Greece	randomised	N = 21 women; 7 with treatment	Not specified	UTI (at least 3 episodes caused by Enterobacteriaceae in the	Nitrofurantoin (100 mg daily for 30 days)	Faecal samples (and urine) cultured and plated on selective	impact of doses of norfloxacin, trimethoprim-sulfamethoxazole and nitrofurantoin	Before antibiotic treatment all stools contained Enterobacteriaceae and *Enterococcus* spp. Nitrofurantoin did not alter abundance of Enterobacteriaceae or *Enterococcus* spp. in the colonic flora during treatment. No

For peer review only - http://bmjopen.bmj.com/site/about/guidelines.xhtml

					preceding 12 months)		agar, API test kits for identification	on aerobic bowel flora	increased resistance to nitrofurantoin in Gram negative aerobic bacteria. No patient receiving the drug was found to be colonised by yeasts.
**Doxycycline**									
**Heimdahl & Nord, 1983[55]**	Sweden	Trial	N = 10	Not specified	Healthy volunteers	Doxycycline 100mg daily for 7 days	Faecal culture on selective media	Effect of doxycycline on the normal human flora and on colonization of the oral cavity and colon	No change in abundance of *Bacteroides* spp., *Bifidobacterium* spp, *Clostridium* spp., *Eubacterium* spp., *Lactobacillus* spp. or *Veillonella* spp. Fusobacteria were eliminated. A 2-3 log decrease in enterococci, streptococci and enterobacteria. Emergence of new strains *Klebsiella pneumoniae*, *Proteus mirabilis* and *Enterobacter cloacae* in some subjects. All colonizing microorganisms were resistant to doxycycline. Normalisation of aerobic bacterial abundance 9 days after antibiotic treatment.
**Walker et al. 2005[56]**	USA	RCT	N = 69 adult; 55 analysed	Clinic	Periodontitis	20 mg doxycycline	Faecal samples plated on a number of selective agar and incubated for CFU counts	To determine if a 9-month regimen of sub optimal doxycycline had an effect on either the intestinal or the	The only statistically significant differences between the two treatment groups occurred in the doxycycline-resistant counts at the baseline sample. No between-treatment differences were detected at 3- or 9-month period either in the predominant bacterial taxa or in antibiotic susceptibilities

For peer review only - http://bmjopen.bmj.com/site/about/guidelines.xhtml

								vaginal microflora	
**Matto et al. 2008[57]**	Finland	Not specified	N = 19; 10 controls	Not specified	Not specified	Doxycycline 150mg daily for 10 days with probiotic	Faecal 16S rRNA PCR for Bifidobacterium sp and DGGE and culturing and sequencing	To evaluate the influence of doxycycline therapy on the composition and antibiotic susceptibility of intestinal bifidobacteria	Bifidobacterium diversity was markedly higher in the control group than antibiotic group; each subject had 2-3 genotypes in the control group; 0-3 in the antibiotic group. The isolated bifidobacteria represented *B. adolescentis/ ruminantium*, *B. longum*, *B. catenulatum/* pseudocatenulatum, *B. bifidum* and *B. dentium*. Tetracycline-resistant Bifidobacterium isolates were more commonly detected in the antibiotic group than in the control group, thus increasing the pool of resistant commensal bacteria in the intestine.
**Rashid et al. 2013[58]**	Sweden	double-blind, randomised, placebo-controlled, parallel group study	N= 34, 17 treated, 17 controls	Clinical trial unit	Healthy volunteers	doxycycline 40 mg capsules orally once daily	Culture on selective media aerobic and anaerobic	primary objective of this study was to assess the impact of antimicrobial treatment on the oropharyngeal and intestinal microflora	Doxycycline was detectable in stool up to 16 weeks. No changes in abundance (>2log CFU/g) of *Bacteroides* spp., *Bifidobacterium* spp., *Clostridium* spp., *Candida* spp., *Lactobacillus* spp., or Enterobacteriaceae anaerobic intestinal microflora. No new colonisation with *C. difficile*. At week 20 the anaerobic microflora was normal. In aerobic intestinal microflora. There were changes (2 log CFU/g) in the numbers of

For peer review only - http://bmjopen.bmj.com/site/about/guidelines.xhtml

								during and after administration of 40 mg doxycycline capsule given once daily. to healthy volunteers.	enterococci and *E. coli* during the 16 weeks treatment. Other microorganisms such as other enterobacteria, *Candida* spp. and other microorganisms were not affected. The aerobic microflora was normal at week 20. Increase in doxycycline resistance in *Bifidobacterium* spp., anaerobic cocci and Gram positive rods.
**Clarithromycin**									
**Brismar et al. 1991[59]**	Sweden	Cohort study	N = 10 adults	Not specified	Healthy volunteers	Clarithromycin 250mg twice daily for 7 days	Culture on selective and non-selective agar	To compare the effect of clarithromycin and erythromycin on the normal flora	Clarithromycin decreased the numbers of *Bacteroides* spp. *Bifidobacterium* spp. *Lactobacillus* spp. And *Streptococcus* spp. Anaerobic cocci increased and *C. albicans* was isolated from 2 patients. No changes in the abundance of *Bacillus* spp., *Clostridium* spp, *Corynebacterium* spp., *Enterococcus* spp., *Eubacterium* spp., *Micrococcus* spp., *Staphylococcus* spp. or *Veillonella* spp. Normalisation in abundance of

For peer review only - http://bmjopen.bmj.com/site/about/guidelines.xhtml

									Enterobacteriaceae, *Lactobacillus* spp. and *Streptococcus* spp. but not of *Bacteroides* spp. and *Bifidobacterium* spp. 14 days after antibiotic treatment
**Edlund et al. 2000**[60]	Sweden	Healthy volunteers	N = 12 male adult	Not specified	Healthy	Clarithromycin 500 mg twice daily for 7 days	Faecal; inhibition assay, culture on selective and non-selective agar	to investigate the ecological effects of moxifloxacin with those of clarithromycin on the intestinal human microflora	Clarithromycin significantly reduced *E. coli* in numbers during treatment but returned to pre-treatment levels at day 35. 6 subjects were colonised by resistant aerobic Gram negative rods, Citrobacter, Klebsiella, Proteus and Pseudomonas. No overgrowth of yeasts. Total number of anaerobic bacteria decreased during treatment. Bifidobacteria were eliminated or strongly reduced. Bacteroides declined. Lactobacilli and clostridia were markedly declined but not significantly. Only minor alterations in numbers of peptostreptococci,

For peer review only - http://bmjopen.bmj.com/site/about/guidelines.xhtml

									veillonellacocci. No overgrowth of *C. difficile*. Bacterial abundance was normalised 28 days after antibiotic treatment
**Edlund et al. 2000**[61]	Sweden	Randomised double blind controlled study	N = 20 adults	Not specified	Healthy	Clarithromycin 500 mg for 10 days	Faecal; culture on selective agar aerobic and anaerobic, Gram stain and biochemical tests, gas liquid chromatography	assess the impact of antibiotic on intestinal microflora	Moderate disturbances in aerobic intestinal microflora, number of *E. coli* significantly reduced at day 10. Overgrowth of *Klebsiella*, *Citrobacter* and *Enterobacter* spp. occurred in 6 subjects receiving clarithromycin. No significant overgrowth of *Candida* spp. There was a marked reduction in *Bifidobacterium* spp. *Clostridia* spp. *Lactobacillus* spp. *Micrococcus* spp. *Staphylococcus* spp. No change in the total numbers of anaerobic bacteria, *Enterococcus* spp., *Peptostreptococcus* spp., *Bacteroides* spp, *Candida* spp., *Streptococcus* spp., or *Veillonella*

For peer review only - http://bmjopen.bmj.com/site/about/guidelines.xhtml

									spp. The lower numbers of *Bifidobacterium* spp. and *Lactobacillus* spp. persisted even after 14 days of no antibiotics
**Matute et al. 2002**[62]	The Netherlands	Double blind randomised trial	N = 18, 6 treatment, 6 controls	Medical Centre	Healthy volunteers	Clarithromycin 500mg twice a day orally for 7 days	Faecal culture on selective agar aerobic and anaerobic	to compare the effect of 3-day, twice daily clarithromycin and with placebo on the faecal microflora	No change in abundance of Candida spp. or Enterococcus spp. No new colonisation with C. difficile or non-fermenters. No increase in clarithromycin-resistant bacteria. The number of organisms of the family Enterobacteriaceae decreased slightly after antibiotic, but levels normalized by day 21 after therapy. The total number of anaerobic bacteria was not affected in the study group.
**<u>Phenoxymethylpenicillin</u>**									
**Heimdahl & Nord 1979**[63]	Sweden	Trial	N = 20, 10 with phenoxymethyl penicillin	Research Institute	Healthy volunteers	Phenoxymethylpenicillin 800 mg as a loading dose, 800 mg twice daily for 7 days	Faecal culture on selective media under aerobic and anaerobic	Effect of phenoxymethylpenicillin (and clindamycin) on the aerobic	No change in abundance of *Acidaminococcus* spp., *Bacteroides* spp., *Bifidobacterium* spp., *Clostridium* spp., Enterobactericaeae,

For peer review only - http://bmjopen.bmj.com/site/about/guidelines.xhtml

							conditions. Bacterial identification by API strips	and anaerobic microflora in the human mouth, throat and colon	*Enterococcus* spp., *Eubacterium* spp., *Fusobacterium* spp., *Lactobacillus* spp., *Megashaera* spp., *Streptococcus* spp. or *Veillonella* spp
**Adamsson et al. 1997**[64]	Sweden	Healthy volunteers	N = 20 adults; 10 with treatment	Not specified	healthy	Phenoxymethylpenicillin 1000 mg twice a day for 10 days	Faecal antimicrobial assay and culture	to investigate the ecological effects of phenoxymethylpenicillin, on the oropharyngeal and intestinal human microflora	No change in abundance of *Bacillus* spp., *Bacteroides* spp., *Enterococcus* spp. or *Streptococcus* spp. There were minor alterations in numbers of *E. coli*. Three subjects became colonised with Klebsiella and one with high numbers of non-fermentative gram-negative rod. Numbers of Clostridium increased. No change in total numbers of aerobic or anaerobic bacteria. Normalisation of bacterial abundance day 14 after antibiotic treatment.
**Erythromycin**									
**Heimdahl & Nord 1982**[65]	Sweden	Trial	N = 10	Research institute	Healthy volunteers	Erythromycin stearate was given orally in doses of	faecal specimens were taken	The impact of erythromycin administration	Suppression of both aerobic and anaerobic faecal flora occurred. All subjects were colonized by

For peer review only

						500 mg twice daily for 7 days	up to 16 days for cultivation of aerobic and anaerobic bacteria	on the normal human flora and on colonization of the oral cavity, throat and colon	erythromycin-resistant enterobacteria, clostridia or yeasts in the colon. Aerobes the number of enterobacteria, enterococci and streptococci were reduced. Anaerobes no change in abundance of *Bifidobacterium* spp, *Eubacterium* spp. or *Lactobacillus* spp. However, Bacteroides, fusobacteria and Veillonella were eliminated in some subjects.
**Brismar et al. 1991**[59]	Sweden	Cohort study	N = 10 adults	Not specified	Healthy	Erythromycin 1000mg twice daily for 7 days	Faecal culture on selective media	Compare the effect of clarithromycin and erythromycin on intestinal microflora	Changes in the intestinal aerobic and anaerobic microflora. Streptococci eliminated, enterobacteria strongly suppressed. Minor reduction in enterococci and corynebacterial. Staphylococci and *C. albicans* increased. Normalisation in abundance of *Bifidobacterium* spp. and *Lactobacillus* spp. 14 days after treatment.

For peer review only - http://bmjopen.bmj.com/site/about/guidelines.xhtml

									Anaerobic, lactobacilli, bifidobacteri, clostridia and bacteriodes reduced. No normalisation in abundance of *Bacteroides* spp. and *Clostridium* spp. 14 days after antibiotic treatment.

For peer review only

For peer review only - http://bmjopen.bmj.com/site/about/guidelines.xhtml

198

199 **Assessment of methodological quality**

200 Selection bias was assessed with the Critical Appraisal Skills Programme (CASP) checklist
201 (www.casp-uk.net). Each component of the studies (i.e., the appropriateness of the study
202 design for the research question, the risk of selection bias, exposure measurement, and
203 outcome assessment) was graded and an overall grading used to produce quality assessment
204 charts based on a traffic light system of 'low', 'high' and 'unclear' or 'not applicable', as
205 recommended by Cochrane.[66] Three reviewers (VW, KE, EvdW) independently assessed the
206 risk of bias in the studies with two reviewers (KE, VW) assessing all studies, and discrepancies
207 were resolved by consensus between the reviewers.

208 **Data synthesis and analysis**

209 Due to the insufficient number of studies available, we were unable to undertake any
210 sensitivity analysis to determine robustness of the findings and the quality of the studies.[66]
211 The results are discussed descriptively.

212 **Patient and Public Involvement**

213 This research was done without patient involvement.

214

For peer review only

For peer review only - http://bmjopen.bmj.com/site/about/guidelines.xhtml

Results

Overall

The database searches returned 2475 articles (May 2020). Following removal of duplicates and screening of records by title or abstract, 2164 were excluded , based on our selection criteria (Figure 1). One hundred and fifty nine full-text articles were evaluated and a further 128 were excluded with reasons detailed in Figure 1. Thirty-one studies were eligible and included in the present review. The included studies are summarised in Table 3 and are briefly described below[31 36-65] Twelve of the studies were published by different authors from the same Swedish study group although one of the studies was carried out in Germany[41-43 50 55 58-61 63-65]

Publication dates ranged from 1979 to 2017. The studies measured the effects on gut microbiota following clinical treatment of the patient or experimental exposure of volunteers to different antimicrobial agents.

The 31 publications involved 1068 patients, although not all of these received the antibiotics of interest and some were comparative control groups. Most studies originated from European countries, Sweden (11), Finland (3), Netherlands (3), France (2) and one each for, Germany, Switzerland, Belgium/Poland and Greece. The remaining studies were conducted in the USA (6), Chile (1) and Brazil (1).

Study Characteristics

Sampling

For peer review only - http://bmjopen.bmj.com/site/about/guidelines.xhtml

235 Faecal samples have generally been accepted as representative of the intestinal microbiota.
236 Studies conducted before 2000, used cultured homogenised faecal samples on non-selective
237 and selective media, to enumerate different colony types. Some isolated pure cultures for
238 identification to genus level. The effect of the antibiotic was measured by comparing the
239 numbers of bacteria present from each genus before treatment with the decrease, increase
240 overgrowth or proliferation of other bacteria. However, it is known that many types of gut
241 bacteria, about 80%, have not been cultured.[67] Predominantly, later studies used more rapid
242 molecular methods.

243 **Analytical techniques**

244 All studies analysed faecal samples, either by culture (17 studies) or molecular techniques (13
245 studies), one study used both methods. Eighteen of the studies used healthy volunteers, 4 of
246 the studies looked at patients with a respiratory infection, 3 studies patients had a urinary
247 tract infection, 1 study reported patients with urinary and respiratory infections, 1 study
248 patients had sinusitis, 1 study they had periodontitis, another studyrelied on self-reported
249 infections and 2 studies did not specify the health status of the subjects. Twelve of the studies
250 were from the same Swedish research institute (although one was conducted in Germany).

251 **Risk of Bias**

252 The risk of bias among the studies was assessed using the Critical Appraisal Skills Programme
253 (CASP) checklists for case controls, randomised controlled trials (RCT), other trials and cohort
254 studies (Figure 2). There was 1 case control appraisal, 15 cohort studies and 15 trials. All the
255 studies adequately stated their study aims and hypothesis and this part of the assessment
256 was rated as 'low' risk.

For peer review only - http://bmjopen.bmj.com/site/about/guidelines.xhtml

Case control

There was 1 case control study included in our review of a patient presenting with onset of diarrhoea not associated with Clostridium infection, within 24 h of antibiotic administration.[48] It was shown that this was associated with changes in the diversity of the gut microbiota. In terms of bias, the study trends towards overall unclear risk as there is lack of controls and the report details just one patient in a clinical setting with a symptom of interest (Figure 2a). However, they did use very robust molecular methods to study these temporal changes in the diversity of the microbiota.

Cohorts

There were fifteen cohort studies included in our review. Assessment relating to the validity of the study are covered within the questions 1-6 (Figure 2b). Generally, these studies were low risk. The reliability of the studies determined by the size of the effect and how precisely this was estimated are covered with questions 7-9 and are low or unclear risk. There is greater unclear risk when it comes to the applicability of the results (questions 10-12), the usefulness for clinical decision making, often the population size was too small to extrapolate results to a larger population as a whole.

Trials

Fifteen studies included in this review were trials/RCTs and overall the risk was more unclear. The validity of the studies (Q1-6), randomisation and masking of treatment allocation was generally low risk. The baseline characteristics of the study were low or unclear risk as most studies stated age, gender and health status, a few exceptions stated BMI and ethnicity. Follow up of study participants was low risk, with most studies taking samples up to 4 weeks

For peer review only - http://bmjopen.bmj.com/site/about/guidelines.xhtml

after antibiotic administration (although there are some indications that this needs to be longer). Controlling for confounding factors (Q6) was unclear in most of the studies. The size of treatment effect (Q7) was well reported but precision (Q8) was unclear, mostly due to small population size and lack of statistical analysis. The transferability of the results to other populations (Q9 and11) were unclear risk.

Antibiotic effects on the gut

The studies analysed, employed different methodologies to explore the effect of antibiotics on the gut microbiota. They have been consistent in demonstrating that dysbiosis develops upon antibiotic administration, which is rapid and results in losses of diversity and shifts in abundance of gut microbes either up or down. After antibiotic treatment, the composition generally returns to a similar pre-treatment state within several weeks, but not in all cases. Here we describe the results in more detail per antibiotic of interest.

Amoxicillin

Ten studies examined the impact of orally administered amoxicillin on the normal intestinal microflora and are summarised in Table 4[36-45]. In brief, administration of amoxicillin leads to an increase or over growth of Enterobacteria (6 studies). The changes in the anaerobic population (includes Lactobacilli, Bifidobacteria and Bacteriodes) varied enormously between studies: from no change, to increases and/or decreases, as well as changes in diversity. There were two reports that isolated Clostridia and Candida and two where no change in numbers were observed. Eight of the studies had some longer term follow up and most of these reported that the populations returned to normal within 2-4 weeks. Three of the studies reported that significant alterations in the microbiome diversity or abundance were still

For peer review only - http://bmjopen.bmj.com/site/about/guidelines.xhtml

301 evident from 42 days to 6 months after the final antibiotic dose. Other longer-term effects 302 were a persistence of Candida at 6 weeks, appearance of other aerobic or resistant Gram 303 negative bacteria (species not specified) at 28 days, and a persistent decrease in 304 *Lachnospiraceae* at 6 months, with no similarities in the populations treated.

For peer review only

For peer review only - http://bmjopen.bmj.com/site/about/guidelines.xhtml

Table 4. Impact of Amoxicillin on the gastrointestinal microflora.

Study	Dose mg/day	Days of administration	Number of patients	Impact on							Overgrowth	
				Aerobic GP cocci	Pseudomonas	Enterobacteria	Anaerobic	Lactobacillus	Bifidobacteria	Bacteriodes	Clostridium	Candida
Christensson et al. 1991[41]	250x3	7	38	No change	isolated 4 patients	Increase		Increase	Increase	Increase	Isolated 2 patients	Isolated 10 patients
Floor et al. 1994[45]	500x3	7	40	Slight change	-	Increase	No change	-	-	-	-	Increase
Mangin et al. 2010[40]	50/kg/d	7	31	-	-	-	-	-	Changes in diversity	-	-	-
Monreal et al. 2005[37]	-	-	22	-	-	-	-	Decrease	Decrease	Decrease	-	-
Ladirat et al. 2014[44]	375x3	5	12	-	-	Overgrowth	-	-	Decrease	-	-	-
Pallav et al. 2014[38]	250x3	7	8	-	-	Increase	Decrease	-	-	Increase	-	-
Brismar et al. 1993[42]	500x3	7	10	Minor decrease	-	Increase	No change	No change	No change	No change	None	No change
Edlund et al. 1994[43]	500x3	7	10	-	-	Overgrowth	-	No change	No change	No change	No change	No change
De La Cochetiere et al. 2005[36]*	500x3	6	5	-	-	-	-	-	-	-	-	-

For peer review only - http://bmjopen.bmj.com/site/about/guidelines.xhtml

							**Fusobacteriaceae**	**Lachnospiraceae**	**Bifidobacteria/Erysipelotrichaceae**	**Bacteriodales/Veillonellaceae/Porphyromonadaceae**		
Abeles et al. 2016[39]†	500x2	3/6	6/6				Increase	Diminished even after 6 mo	Decrease then increase	Decrease		

Notes

- = not reported

* this paper measured similarity indices of TGGE profiles and showed shifts in dominant species upon antibiotic treatment

† also a reduction in diversity

GP = Gram positive

For peer review only

For peer review only - http://bmjopen.bmj.com/site/about/guidelines.xhtml

Christensson *et al.* [41] conducted a culture dependent randomised study of 84 subjects with lower respiratory tract infections, 38 of whom completed treatment with amoxicillin (the remaining received cefaclor and will not be discussed further here). They showed that the numbers of enterobacteria, anaerobic Gram-positive Lactobacilli, Bifidobacteria and Eubacteria, and anaerobic Bacteroides increased significantly in the amoxicillin group. The number of anaerobic cocci was reduced in 18 patients and increased in 11 patients. There was no change in the abundance of *Enterococcus* spp., *Staphylococcus* spp. or *Streptococcus* spp. Four patients became colonised with *Pseudomonas* sp. and five with *C. albicans*. Although *C. albican*s was also isolated from two patients pre-treatment and three patients six weeks after treatment. *C. difficile* was isolated from two patients during treatment.

The effect of amoxicillin on the intestinal microflora of patients with bronchitis was evaluated in a randomised study by Floor *et al.* [45]. The anaerobic flora remained unchanged, both during, and after therapy with amoxicillin. The total number of aerobic Gram-negative rods increased significantly and in 37.5% of the patients with newly acquired bacteria the main species identified were *Klebsiella*. Enterococci were cultured in only one patient. A third of patients had other aerobic bacteria different to baseline on days 21-28. Bacterial and fungal abundance returned to baseline levels 21 days after treatment.

Mangin *et al.* [40] studied 18 month old children with acute bronchitis. Both total bacteria and numbers of Bifidobacteria were similar during the course of antibiotic treatment. However, changes in Bifidobacterium at the species level showed decreased diversity. Amoxicillin treatment induced a complete disappearance of *Bifidobacterium adolescentis* species, a significant decrease in the occurrence rate of *Bifidobacterium bifidum*, but did not affect

For peer review only - http://bmjopen.bmj.com/site/about/guidelines.xhtml

336 *Bifidobacterium longum* and *Bifidobacterium pseudocatenulatum/Bifidobacterium*
337 *catenulatum*. They did not study longer term effects.

338 Monreal *et al.* [37] used culture based methods to examine the populations of faecal bacteria
339 in 22 patients with respiratory tract infections treated with amoxicillin. *Bacteroides* spp.,
340 *Bifidobacterium* spp. and *Lactobacillus* spp. were decreased with antibiotic treatment.
341 However, this was only transient and thirty days after the end of treatment the levels of
342 Bifidobacterium and Lactobacillus had recovered to their normal values. Bacteroides were
343 slower to recover.

344 Ladirat *et al.* [44] studied a small number of healthy volunteers with amoxicillin and a prebiotic
345 specifically looking at total bacteria and Bifidobacterium. Numbers of Bifidobacterium
346 decreased over time due to amoxicillin treatment especially in the group with no prebiotic.
347 Amoxicillin affected the abundance and diversity of *Bifidiobacterium* spp. and there was an
348 overgrowth of Enterobacteriaceae. The observations were highly individual dependent. In
349 the follow-up period (3 weeks after the discontinuation of amoxicillin treatment), the levels
350 of bifidobacterial in faeces returned to the initial levels.

351 Amoxicillin was administered randomly to a small number of healthy volunteers and the
352 impact on intestinal microflora (and oropharyngeal) was studied by.[42] In the aerobic intestinal
353 flora, a minor decrease in the numbers of streptococci and staphylococci was observed.
354 Overgrowth of Klebsiella species was seen in six subjects and of Enterobacter species in two
355 subjects. No overgrowth of enterococci or yeasts occurred. In the anaerobic microflora only
356 the number of eubacteria was reduced. There was no colonisation by *C. difficile*. Two weeks
357 after administration of amoxicillin the microbiota had returned to baseline levels.

For peer review only - http://bmjopen.bmj.com/site/about/guidelines.xhtml

358 Another study on the effect of amoxicillin on intestinal microflora of healthy volunteers showed only small alterations in the aerobic faecal microflora.[43] All major anaerobic bacterial groups were unaffected by amoxicillin and there was no colonisation by *C. difficile*. There was and overgrowth of *Klebsiella* spp. in 6 subjects and Enterobacter in 2 subjects. Intestinal microflora returned to normal 2 weeks after treatment had been discontinued.

363 Six healthy volunteers received oral amoxicillin for 5 days and their faecal microbiota examined by 16S rRNA gene amplification and gradient gel electrophoresis until 60 days.[36] Dominant species diversity profiles were compared on the basis of similarity with percentages on first sampling ranging from 93% to 99%. During the 5 day course of amoxicillin treatment these percentages decreased to an average of 73%, but there was huge variation between individuals. By day 60, 5 of the 6 subject gel profiles had returned to their near initial composition. Gel banding patterns were examined in more detail for 3 individuals and were shown to correspond to *Clostridium nexile*, *Ruminococcus torques* and β-*Proteobacteria*.

371 Abeles *et al.* [39] studied cohabiting individuals over 6 months where one was given an antibiotic and the other a placebo. The microbiota of subjects taking amoxicillin grew more dissimilar over time although not statistically significant, as similar trends were also seen in those subjects taking placebo. Taxonomic compositions of the gut differed after amoxicillin therapy. *Lachnospiraceae* were significantly diminished and remained diminished at 6 months. Veillonellaceae, Bacteroidales, and Porphyromonadaceae were significantly decreased in response to amoxicillin, while Fusobacteriaceae were increased. Bifidobacteriales and Erysipelotrichaceae were initially decreased, and subsequently increased in comparison to their housemates taking placebo.

For peer review only - http://bmjopen.bmj.com/site/about/guidelines.xhtml

Pallav et al.[38] studied the effect of amoxicillin treatment with and without a prebiotic. Eight subjects that received just the antibiotic had substantial microbiome changes most notably an increase in Escherichia/Shigella. There was no change in abundance in the control group. Antibiotic associated changes persisted to the end of the study, 42 days after antibiotic therapy ended.

Amoxicillin with clavulanic acid

Seven studies have looked at the impact of amoxicillin in combination with clavulanic acid on the gut microbiota.[31 46-51] These are summarised in Table 5. Briefly, the combination of amoxicillin with clavulanic acid caused, mild to moderate changes in microbiome composition, mainly increases in Enterobacteria with varying effects on anaerobic bacteria *Bifidobacterium* sp., *Lactobacillus* sp. and *Bacteriodes* sp. and a general decrease in diversity. One study reported that bacterial abundance was not normalised 2 months after antibiotic treatment was stopped, and four reported levels similar to baseline within 35 days..

For peer review only

For peer review only - http://bmjopen.bmj.com/site/about/guidelines.xhtml

Table 5. Impact of Amoxicillin with Clavulanic acid on the gastrointestinal microflora.

Study	Dose mg/day	Days of administration	Number of patients	Impact on							Overgrowth	
				Aerobic GP cocci	Pseudomonas	Enterobacteria inc. *E. coli*	Anaerobic cocci	Lactobacillus	Bifidobacteria	Bacteriodes	Clostridium	Candida
Lode et al. 2001[50]	875/125	7	12	Increase	-	Increase	Little change	Decrease	Decrease	Little change	Decrease	
Young & Schmidt 2004[48]*	875/125x2	10	1	-	-	Clusters present	-	-	Clusters present	Clusters present	Two clusters	
Engelbrektson et al. 2009[49]†	875/?x2	7	32	-	-	Increase	-	No trend	No trend	Increase	No trend	
Forssten et al. 2014[47]†	875/125	7	80			Increased		Not affected in probiotic group	Decreased in the placebo group		Decreased	
Korpela et al. 2016[31]**	-	-	142					Slight decrease		Two fold elevated Parabacteroides		
Kabbani et al. 2017[51]	875/125x2	7	12	Increase		Increased		Decreased	Decreased	Increased	Decreased	
Mangin et al. 2012[46]	875/125x2	5	18			Increased			Decreased			

For peer review only

*this paper reported shifts in the representation of the major bacterial groups

†probiotic also given

** and penicillin V

GP = Gram positive

For peer review only

For peer review only - http://bmjopen.bmj.com/site/about/guidelines.xhtml

Administration of amoxicillin/clavulanic acid to healthy people caused increased numbers of enterococci and *E. coli* strains in the aerobic microflora while Bifidobacteria, Lactobacilli, and Clostridia decreased significantly.[50] Numbers of anaerobic cocci and Bacteriodes were not markedly altered. *C. difficile* strains were recovered from three of the volunteers. The microflora was normalised in all volunteers after 35 days.

Young and Schmidt[48] investigated the short-term impact of amoxicillin/clavulanic acid prescribed for a male patient with acute sinusitis, who developed antibiotic-associated diarrhoea. It was shown that the major bacterial groups were partially restored 14 days after antibiotic treatment, except for Bifidobacterium. During antibiotic administration no sequences corresponding to butyrate-producing *Clostridium* cluster XIVa were detected, but two weeks after cessation of antibiotics there was a reappearance of this cluster. In this case study the decrease in this cluster may be linked to the antibiotic-associated diarrhoea.

One arm of a study by Engelbrektson *et al.*[49] observed the effects of probiotic treatment concurrent with antibiotic therapy on faecal communities. They analysed their data by measuring individual divergences from baseline levels after treatment to address the problem of subject-to-subject variability. Subjects fell into two categories those with a stable baseline microbiota and those where it varied significantly. Culture data showed increasing numbers of Bacteroides and Enterobacteriaceae, but no trend for Clostridium, Bifidobacterium and Lactobacillus. The antibiotic group (without probiotic) had a significant change in numbers of bacteria from baseline at day 21.

For peer review only - http://bmjopen.bmj.com/site/about/guidelines.xhtml

Forssten *et al.*[47] also studied antibiotic administration in combination with probiotic. Consumption of the probiotic combination mainly lead to an increase in the faecal levels of the species included in the preparation. The antibiotic had only minor effects on Lactobacillus, Bifidobacterium, Bacteroides, Enterobacteriaceae and Clostridium.

Korpela *et al.*[31] looked at the macrolides amoxicillin with and without clavulanic acid and penicillin V. Macrolide use reduced abundance of Actinobacteria and increased Bacteriodetes and Proteobacteria. Penicillin groups did not have distinctly different phyla composition. Firmicutes was reduced. The effect of macrolide use was long lasting and associated with increased risk of asthma and antibiotic-associated weight gain.

Twelve healthy subjects received amoxicillin/clavulanate for 7 days and their stool specimens were analyzed using 16s rRNA gene pyrosequencing.[51] Antibiotic associated changes included reduced prevalence of the genus *Roseburia* and increases in *Escherichia*, *Parabacteroides*, and *Enterobacter*. Microbiota alterations reverted toward baseline, but were not completely restored 2 weeks after treatment.

Real-time polymerase chain reaction (PCR) with temporal temperature gradient gel electrophoresis (TTGE) showed that at the end of a 5 day course of amoxicillin-clavulanic acid total bacterial and Bifidobacteria concentrations were significantly reduced in 18 healthly volunteers.[46] At the same time, the mean similarity percentages profiles were significantly reduced and the number of distinct Bifidobacterium species per sample significantly decreased. Two months after antibiotic exposure, the mean similarity percentage had not normalised.

Nitrofurantoin

For peer review only - http://bmjopen.bmj.com/site/about/guidelines.xhtml

443 Three studies investigated the effect of nitrofurantoin macrocrystals on faecal microbiota compositions.[52-54] Two studies used 16S rRNA gene sequencing and one study bacterial culture and these are summarised in Table 3. Nitrofurantoin inhibits bacterial DNA, RNA, and cell wall protein synthesis. It is used prophylactically as a urinary anti-infective agent against most gram-positive and gram-negative organisms and for long-term suppression of infections. Upon oral administration, most of the nitrofurantoin is rapidly absorbed in the small intestine and eliminated primarily by kidney glomerular filtration into urine, where it reaches higher and more effective therapeutic concentrations. Only a small amount reaches the colon, which may account for the minor impact on intestinal microflora.

452 One of the studies investigated a small population of women (n=7) with recurrent urinary tract infections and it showed that nitrofurantoin did not alter either the Enterobacteria, Enterococci or yeasts of the colonic flora during treatment[54]. No resistant strains of Gram negative aerobic bacteria were detected.

456 Stewardson *et al.*[52] treated UTI patients (n=10) with nitrofurantoin macrocrystals and showed that it was not associated with a statistically significant global impact on the gut microbiota (weak effect). Nitrofurantoin treatment was associated with an increase in the proportion of the genus Faecalibacterium and a decrease in the proportion Clostridium (Clostridiaceae) at the end of the antibiotic treatment. Another small study (n=8) treated uncomplicated UTIs and did not show any significant impact of nitrofurantoin treatment on the faecal microbiota other than a temporary increase in the Actinobacteria phylum, more specifically in the beneficial Bifidobacterium genus.[53] Bacterial abundance had returned to pre-antibiotic levels 31 to 43 days after stopping antibiotic treatment, while the other study did not do follow-up testing.[52 53]

For peer review only - http://bmjopen.bmj.com/site/about/guidelines.xhtml

Doxycycline

Four studies reported on the effect of doxycycline on faecal microbiota, one at sub optimal dosage (20mg for 9 months) in patients with periodontitis[56] and two at usual dose 100-150mg for 7-10 days, although one also with a probiotic[55 57], and another at low dose (40mg for 16 weeks),[58] as described in Table 3. Doxycycline treatment did not significantly affect counts of total anaerobic bacteria, candida, total enterics, Staphylococcus or doxycycline-resistant bacteria recovered at any of the sample periods and did not result in the development of multi-antibiotic resistance.[56] Matto *et al.*[57] specifically evaluated the influence of doxycycline therapy on the composition and antibiotic susceptibility of intestinal Bifidobacteria in nine subjects whilst they were also taking a probiotic and compared these to adults consuming only probiotics. A marked decrease in diversity of Bifidobacterium populations was observed during doxycycline therapy. Tetracycline-resistant Bifidobacterium isolates were more commonly detected in the antibiotic group than in the control group, thus increasing the pool of resistant commensal bacteria in the intestine.

In another study on 10 healthy volunteers, doxycycline decreased the abundance of Enterobacteriaceae, *Enterococcus* spp., *E. coli*, and *Streptococcus* spp.[55] With the exception of *Fusobacterium* spp., which was eliminated, the number of anaerobic bacteria in faeces was not influenced by doxycycline.[55] After doxycycline administration, bacterial abundance was reported to have returned to pre-antibiotic levels 9 days after stopping treatment.[55] The fourth study also looked at a low dose over a period of 16 weeks.[58] There were 2 log decreases in numbers enterococci and *E. coli*. Other aerobic microorganisms including enterobacteria, *Candida* spp. were not affected. There were no significant changes in numbers of anaerobic lactobacilli, bifidobacteria, clostridia and Bacteroides during doxycycline administration.[58] No

For peer review only - http://bmjopen.bmj.com/site/about/guidelines.xhtml

C. difficile strains were isolated. The aerobic and anaerobic microflora was normal at 28 days after stopping treatment.[58]In summary, doxycycline interferes with a microorganism's ability to manufacture proteins. At suboptimal dosage it has little effect on the gut microbiota with the exception of enterococci and E. coli. At normal dosage it markedly affects the diversity of Bifidobacteria populations in one study and eliminated Fusobacterium sp. in another. The effect on populations of other gut bacteria seem transient and normalisation was reported by 28 days. Longer term effects are not known.

Clarithromycin

There were four articles on influence of clarithromycin included. Three were from the same research institute, and these examined the intestinal microflora of healthy volunteers before, during and after administration of clarithromycin. The study design was similar in all three studies and involved small numbers, 10 (a mix of male and female subjects) in two studies[59 61] and 12 men only[60] as described in Table 3. Dosing and faecal sampling strategy and analysis were also similar, 250 mg twice daily for 7 days[59]; 500mg twice daily for 7 days[60] and 250 mg twice daily for 10 days,[61] sampling 3 times during and 3-4 times after administration up to 35 days. The male only subjects were administered another antibiotic with a 6 week wash out period, it is not clear whether this was before or after clarithromycin administration.

The main impact in the earlier study was a reduction in the numbers of Enterobacteria and Streptococci. Lactobacilli, Bifidobacteria and Bacteroides were suppressed in the anaerobic microflora.[59]

Clarithromycin caused a significant reduction of *E. coli* during 7 days of treatment but levels returned to normal 28 days after the end of the course.[60] Six subjects were colonised by

For peer review only - http://bmjopen.bmj.com/site/about/guidelines.xhtml

For peer review only

511 resistant aerobic Gram negative rods, Citrobacter, Klebsiella, Proteus and Pseudomonas. The
512 total numbers of anaerobic bacteria decreased. Bifidobacteria and Bacteriodes were
513 significantly reduced and Lactobacilli and Clostridia were suppressed but this was not
514 significant. There was no overgrowth of *C. difficile* or yeasts. The microflora returned to
515 normal in all subjects after 35 days.

516 In the final study, the numbers of *E. coli* were significantly reduced and in six individuals
517 overgrowth of Klebsiella, Citrobacter and Enterobacter spp. occurred.[61] The total number of
518 anaerobic intestinal bacteria was not affected but the numbers of Lactobacilli and
519 Bifidobacteria were significantly reduced which persisted for the duration of the study. There
520 was no significant overgrowth of *Candida* spp. and no subjects were colonised by *C. difficile*.

521 A fourth study from a different group on 6 healthy volunteers, showed no change in the
522 abundance of *Candida* spp., *Enterococcus* spp. or anaerobic bacteria. There was no new
523 colonisation by *C. difficile*. Numbers of Enterobacteriaceae decreased slightly. Levels were
524 reported to have returned to baseline by day 21 after the course of clarithromycin.

525 To summarise, all four studies reported reduction in Enterobacteria and three reported
526 suppression of anaerobic bacteria after administration of clarithromycin. This reduction was
527 transient for most species except Lactobacilli and Bifidobacteria. Only one studied reported
528 isolation of *C. albicans.*[59] None reported new colonisation or overgrowth of *C. difficle*. All reported
529 normalisation to baseline levels within 28 days of finishing antibiotic treatment.

530 **Phenoxymethylpenicillin**

531 Phenoxymethylpenicillin did not have much effect on gut microbiome as shown by two
532 studies that investigated the effect on the oropharyngeal and intestinal microflora of healthy

For peer review only - http://bmjopen.bmj.com/site/about/guidelines.xhtml

533 volunteers, (Table 3).[63 64] A very early study reported no effect of phenoxymethylpenicillin on
534 abundance of various species comprising the aerobic and anaerobic flora of faecal samples.[63] A later
535 study showed no significant alterations in the total aerobic and anaerobic of the intestinal
536 microflora were observed although three volunteers became newly colonised with *Klebsiella*
537 sp. and one harboured high numbers of a non-fermentative gram-negative rod. The numbers
538 of viridans streptococci, enterococci and Bacillus were unaffected by the administration of
539 phenoxymethylpenicillin, while minor alterations were noticed in the numbers of *E. coli*. The
540 median values of Clostridium species increased during administration, while the numbers of
541 Bacteroides species were unaffected during the study period. The microflora became
542 normalised 2 weeks after withdrawal of the drugs.

543 **Erythromycin**

544 In an early study,[65] it was shown that the administration of 500 mg of erythromycin twice
545 daily for 7 days to 10 volunteers resulted decreased abundance of both aerobic and anaerobic
546 faecal flora. In addition, potentially pathogenic erythromycin-resistant enterobacteria,
547 clostridia or yeasts colonized all subjects.[65] Brismar and colleagues[59] administered
548 erythromycin orally for 7 days to 10 volunteers and evaluated its effect on the colonic
549 bacteria. They found decreases in the numbers of streptococci, enterococci, and
550 enterobacteria during administration, increases in staphylococci, and alteration of the
551 anaerobic bacteria.

552 **Discussion**

553 Main findings

For peer review only - http://bmjopen.bmj.com/site/about/guidelines.xhtml

This systematic review examined the use of the most commonly prescribed antibiotics in primary care to treat respiratory and urinary tract infections and their impact on gut microbiota. Firstly, the studies showed that antibiotics have an impact on the abundances of the bacteria in the gut community, causing rapid and diminished levels of bacterial diversity and taxonomic richness, increases and decreases in the relative abundances of certain taxa, leading to dysbiosis. As well as antibiotic and individual host-specific effects.

Secondly, once treatment has stopped, there was some, but poor, evidence that the gut bacteria show resilience and are capable of some degree of recovery, in most individuals to their initial state. However, the microbiota is often not totally recovered, suggesting some antibiotics have a persistent effect on certain species. These observations underline the importance of restrictive and proper use of antibiotics in order to prevent long-term ecological disturbances of the indigenous microbiota.

Comparative observations

Other reviews have also reported and summarised how antibiotics change the abundance and diversity of the intestinal microbiota.[68-70] Here we focus on those antibiotics routinely prescribed by general practitioners (GPs) in primary care. Often GPs inappropriately prescribe antibiotics for infections caused by viruses, or prescribe a broad spectrum antibiotic, when an antibiotic for a specific bacteria should be used. They may also prescribe the incorrect dose or for the wrong length of time. Health professionals are concerned antibiotics are used too often and incorrectly, which contributes to antimicrobial resistance and with the additional disruption to the microbiome also can have other negative (long term) health effects, such as metabolic and immune disorders.[71 72] These additional effects will further impact on the patient and potentially on the primary care burden. Inappropriate prescribing is due to many

For peer review only - http://bmjopen.bmj.com/site/about/guidelines.xhtml

factors including patients who insist on antibiotics, GPs who do not have enough time to explain why antibiotics are not necessary those who do not know how to recognise a serious bacterial infection, or those who are overly cautious.

Quantifying antibiotic therapy effect on gut flora is challenging. Although the literature describes 'normal', it really means those commensal species whichh are consistently and predominantly found in the healthy human gut. Studies quantifying changes in flora would be easier to interpret if a baseline populations were established in each case. We found a lack of large-scale trials (including RCTs) and observational studies and heterogeneity regarding methodology and outcomes. The heterogeneity is apparent by the inconsistency in the dosage, duration of treatment and follow up, selection and spectrum of antibiotics used, all contributing to varying faecal concentrations. The health status and age of the participant also have an effect, most studies did not clearly define baseline characteristics which causes problems with comparing outcomes. The majority of studies did not report adverse events or any other factors which might affect the efficacy of the antibiotic which in turn might influence the outcome on the microbiome. The studies included used different methods, for example, random allocation of antibiotics to healthy volunteers, or non-randomised treatment where all healthy subjects in the study received antibiotics, also some studies participants were masked to which treatment they received and others were not. There were differences between studies in the way outcomes are defined and measured, for example, culturing versus molecular techniques. These differing analytical techniques may lead to differences in the observed intervention effects. A number of the included studies originate from one research group which could add additional bias to the outcomes. The different classes of antibiotics also have different effects on the microbiome, making it difficult to compare across studies.

For peer review only - http://bmjopen.bmj.com/site/about/guidelines.xhtml

601 The composition of the gut microbiota among humans varies considerably from subject to
602 subject. Grouping of data from several individuals can result in loss of statistical significance.
603 The studies do not consider any confounding factors such as diet, age of subjects, childhood
604 exposure to antibiotics, geographical location, stress or taking probiotic supplements, all of
605 which can complicate the specific antibiotic treatment effects on the gut microbiome profile.
606 These factors can change over a lifetime.

607 Strengths and limitations

608 The primary strength of this study is our focus on the most commonly prescribed antibiotics
609 in primary care and their effect on gut microbiota. Information on how these commonly
610 prescribed antibiotics affect our gut microbiome could influence GP decision making when
611 prescribing antibiotics.. Also, the adverse impact of antibiotics on the gut microbiome is of
612 great importance because when disrupted it can be associated with a variety of diseases,
613 including susceptibilty to infections, autoimmune diseases (such as inflammatory bowel
614 disease), diabetes, depression and obesity. The main limitations of this review are that the
615 majority of the studies included were small, not properly randomised, nor were they large
616 observational studies. The considerable heterogeneity is prone to biases and confounding
617 factors.

618 Inherent in many studies that address the impact of antibiotics on the intestinal microbiota
619 are the limitations of techniques available for analysis. Many have been performed using
620 laborious culture-based methodology which do not lend themselves to analysis of large
621 numbers of samples. The older studies included in our analysis all used culture-based
622 techniques to examine gut microbiota composition, which increases the risk of detection bias.
623 The disadvantage of using culturing is that despite the use of specific selective media and

For peer review only - http://bmjopen.bmj.com/site/about/guidelines.xhtml

anaerobic incubation conditions, there remains a substantial part of the microbiota, approximately 80%,[67] that has not yet been cultured. Culture media choice and sample handling can also skew the data.

The limitations of culture-based techniques can be largely overcome by using molecular approaches.[73] These methods are based on 16S rRNA gene amplification and give us a broader less biased view of gut bacterial composition. The amplified genes are characterised by methods, such as terminal-restriction fragment length polymorphism, denaturing or temperature gradient gel electrophoresis, deep shotgun metagenome and full-length sequencing, some of which are used in the more recent papers in our review. These bioinformatic techniques also have limitations in that they are often more difficult to understand and interpret. The use of different techniques makes it more difficult to compare results and generalisation of conclusions is a result of this diversity in determination and quantification.

Conclusions

The widespread and often overuse of antibiotics has led to the establishment of antibiotic-resistant bacteria and the transfer of resistance genes, which has become a global challenge for infection control and the untreatable nature of bacterial infections. Almost 80% of the antibiotics prescribed within the NHS are being within primary care[1]. The effects of excessive antibiotic exposure can also be seen in the symbiotic microbiotas of the human body.[74] As a result, the microbiota imbalances caused by antibiotics can negatively affect health by increasing susceptibility to infections, compromising immune homeostasis (indicative in increased allergies), asthma, seen by obesity, metabolic syndrome and diabetes.[75]

For peer review only - http://bmjopen.bmj.com/site/about/guidelines.xhtml

Antibiotics have an impact on the gut microbiota causing rapid and diminished levels of bacterial diversity and increases and decreases in the relative abundances, leading to dysbiosis. Once treatment has stopped, there was some evidence that the gut bacteria are capable of some degree of recovery, in most individuals, to their baseline state. The studies do not consider that recovery of the microbiome after antibiotic therapy in the elderly may be affected by age-associated physiological alterations, and other drug-drug interactions.

This systematic review highlights the lack of research and understanding of the effects of commonly prescribed antibiotics on the gut microbiome and shows it is an area which requires studies on larger populations with extended sampling to understand the long-term impacts. Embedding of this knowledge in antimicrobial stewardship programs in primary care will be essential.

Footnotes

Contributors: KE and VW performed the searches. VW, EvdW, AH, LD and KE identified eligible studies. KE, VW and EvdW authors appraised study quality and extracted data. KE and VW drafted first sections of the text and KE completed data analysis, text and tables to circulate to all authors for their contribution to the final draft. ALH advised on search strategy and RoB and read and contributed to the draft. ADH was involved in the main concept, advised on the antibiotics includeed and read and contributed to the draft. EvdW is the guarantor and attests that all listed authors meet authorship criteria and that no others meeting the criteria have been omitted. All authors had full access to the data in the study and can take responsibility for the integrity of the data and the accuracy of the data analysis.

Funding: This research was supported by the NIHR Health Protection Research Unit in Evaluation of Interventions at the University of Bristol. The views expressed in this article are

For peer review only

For peer review only - http://bmjopen.bmj.com/site/about/guidelines.xhtml

those of the author(s) and not necessarily those of the NHS, the NIHR, or the Department of Health and Social Care.

Competing interests: All authors have completed the ICMJE's unified disclosure form at http://icmje.org/conflicts-of-interest/ and declare: all authors had financial support from the University of Bristol for the submitted work; no financial relationships with any organisations that might have an interest in the submitted work in the previous three years; no other relationships or activities that could appear to have influenced the submitted work."

Ethical approval: Not required.

Transparency declaration: Dr Esther van der Werf (the manuscript's guarantor) affirms that the manuscript is an honest, accurate, and transparent account of the study being reported; and that no important aspects of the study have been omitted.

Copyright: Dr Esther van der Werf has the right to grant on behalf of all authors and does grant on behalf of all authors, an exclusive licence on a worldwide basis to the BMJ Publishing Group Ltd to permit this article (if accepted) to be published in BMJ editions and any other BMJPGL products and sublicences such use and exploit all subsidiary rights, as set out in our licence.

Data sharing: if necessary data can be shared.

For peer review only - http://bmjopen.bmj.com/site/about/guidelines.xhtml

References

1. PHE. English surveillance programme for antimicrobial utilisation and resistance (ESPAUR) London: Public Health England, 2018:https://assets.publishing.service.gov.uk/government/uploads/system/uploads/attachment_data/file/759975/ESPAUR_2018_report.pdf.

2. Protection H. Tackling antimicrobial resistance 2019-2024: The UK's 5-year action plan, 2019:1-91.

3. Dolk FCK, Pouwels KB, Smith DRM, et al. Antibiotics in primary care in England: which antibiotics are prescribed and for which conditions? *J Antimicrob Chemoth* 2018;73(suppl_2):ii2-ii10. doi: 10.1093/jac/dkx504 %J Journal of Antimicrobial Chemotherapy

4. Ashworth M, Charlton J, Ballard K, et al. Variations in antibiotic prescribing and consultation rates for acute respiratory infection in UK general practices 1995-2000. *Brit J Gen Pract* 2005;55(517):603-08.

5. Stanton N, Francis NA, Butler CC. Reducing uncertainty in managing respiratory tract infections in primary care. *British Journal of General Practice* 2010;60(581):e466-75.

6. Howie JG. Clinical judgement and antibiotic use in general practice. *British Medical Journal* 1976;2(6043):1061-4.

7. Lindbaek M. Prescribing antibiotics to patients with acute cough and otitis media. *British Journal of General Practice* 2006;56(524):164-6.

8. Tan T, Little P, Stokes T. Antibiotic prescribing for self limiting respiratory tract infections in primary care: summary of NICE guidance. *BMJ* 2008;337

9. Juszczyk D, Charlton J, McDermott L, et al. Electronically delivered, multicomponent intervention to reduce unnecessary antibiotic prescribing for respiratory infections in

For peer review only - http://bmjopen.bmj.com/site/about/guidelines.xhtml

primary care: a cluster randomised trial using electronic health records—REDUCE Trial study original protocol. *BMJ Open* 2016;6(8) doi: 10.1136/bmjopen-2015-010892

10. Car J. Urinary tract infections in women: diagnosis and management in primary care. *Bmj* 2006;332 doi: http://dx.doi.org/10.1136/bmj.332.7533.94

11. Mangin D. Urinary tract infection in primary care. *Bmj* 2010;340 doi: 10.1136/bmj.c657

12. The Human Microbiome Project C, Huttenhower C, Gevers D, et al. Structure, function and diversity of the healthy human microbiome. *Nature* 2012;486:207. doi: 10.1038/nature11234. https://www.nature.com/articles/nature11234#supplementary-information

13. Hollister EB, Gao C, Versalovic J. Compositional and functional features of the gastrointestinal microbiome and their effects on human health. *Gastroenterology* 2014;146(6):1449-58. doi: 10.1053/j.gastro.2014.01.052 [published Online First: 2014/01/29]

14. Jandhyala SM, Talukdar R, Subramanyam C, et al. Role of the normal gut microbiota. *World journal of gastroenterology* 2015;21(29):8787-803. doi: 10.3748/wjg.v21.i29.8787 [published Online First: 2015/08/07]

15. Lloyd-Price J, Abu-Ali G, Huttenhower CJGM. The healthy human microbiome. 2016;8(1):51. doi: 10.1186/s13073-016-0307-y

16. Tuddenham S, Sears CL. The intestinal microbiome and health. *Current opinion in infectious diseases* 2015;28(5):464-70. doi: 10.1097/QCO.0000000000000196

17. Langdon A, Crook N, Dantas G. The effects of antibiotics on the microbiome throughout development and alternative approaches for therapeutic modulation. *Genome Medicine* 2016;8(1):39.

For peer review only

For peer review only - http://bmjopen.bmj.com/site/about/guidelines.xhtml

18. Denny JE, Powell WL, Schmidt NW. Local and Long-Distance Calling: Conversations between the Gut Microbiota and Intra- and Extra-Gastrointestinal Tract Infections. *Frontiers in Cellular & Infection Microbiology* 2016;6:41.

19. Tremlett H, Bauer KC, Appel-Cresswell S, et al. The gut microbiome in human neurological disease: A review. *Annals of Neurology* 2017;81(3):369-82. doi: 10.1002/ana.24901

20. Lurie I, Yang YX, Haynes K, et al. Antibiotic exposure and the risk for depression, anxiety, or psychosis: a nested case-control study. *Journal of Clinical Psychiatry* 2015;76(11):1522-8.

21. Cox LM, Blaser MJ. Antibiotics in early life and obesity. *Nature Reviews Endocrinology* 2015;11(3):182-90.

22. Taur Y, Pamer EG. Microbiome mediation of infections in the cancer setting. *Genome Medicine* 2016;8(1):40.

23. Blaser MJ. Antibiotic use and its consequences for the normal microbiome. *Science* 2016;352(6285):544-5.

24. Ahmad OF, Akbar A. Microbiome, antibiotics and irritable bowel syndrome. *British medical bulletin* 2016;120(1):91-99.

25. Mikkelsen KH, Allin KH, Knop FK. Effect of antibiotics on gut microbiota, glucose metabolism and body weight regulation: a review of the literature. *Diabetes, obesity & metabolism* 2016;18(5):444-53. doi: https://dx.doi.org/10.1111/dom.12637

26. Li M, Wang M, Donovan SM. Early development of the gut microbiome and immune-mediated childhood disorders. *Seminars in Reproductive Medicine* 2014;32(1):74-86.

For peer review only - http://bmjopen.bmj.com/site/about/guidelines.xhtml

27. Lankelma JM, Nieuwdorp M, de Vos WM, et al. The gut microbiota in internal medicine: implications for health and disease. *Netherlands Journal of Medicine* 2015;73(2):61-8.

28. Turta O, Rautava S. Antibiotics, obesity and the link to microbes - what are we doing to our children? *BMC Medicine* 2016;14:57.

29. Cassir N, Simeoni U, La Scola B. Gut microbiota and the pathogenesis of necrotizing enterocolitis in preterm neonates. *Future Microbiol* 2016;11(2):273-92. doi: 10.2217/fmb.15.136 [published Online First: 2016/02/09]

30. Yatsunenko T, Rey FE, Manary MJ, et al. Human gut microbiome viewed across age and geography. *Nature* 2012;486(7402):222-7. doi: 10.1038/nature11053 [published Online First: 2012/06/16]

31. Korpela K, Salonen A, Virta LJ, et al. Intestinal microbiome is related to lifetime antibiotic use in Finnish pre-school children. *Nature communications* 2016;7:10410.

32. Moher D, Liberati A, Tetzlaff J, et al. Preferred reporting items for systematic reviews and meta-analyses: the PRISMA statement. *PLoS Med* 2009;6(7):e1000097. doi: 10.1371/journal.pmed.1000097 [published Online First: 2009/07/22]

33. McDonald LC. Effects of short- and long-course antibiotics on the lower intestinal microbiome as they relate to traveller's diarrhea. *Journal of travel medicine* 2017;24(suppl_1):S35-S38. doi: https://dx.doi.org/10.1093/jtm/taw084

34. Duranti S, Lugli GA, Mancabelli L, et al. Prevalence of Antibiotic Resistance Genes among Human Gut-Derived Bifidobacteria. *Applied and environmental microbiology* 2017;83(3) doi: https://dx.doi.org/10.1128/AEM.02894-16

35. Excellence NIfHaC. Urinary tract infections in adults. London: NICE, 2015.

For peer review only

For peer review only - http://bmjopen.bmj.com/site/about/guidelines.xhtml

36. De La Cochetiere MF, Durand T, Lepage P, et al. Resilience of the dominant human fecal microbiota upon short-course antibiotic challenge. *Journal of Clinical Microbiology* 2005;43(11):5588-92.

37. Monreal MT, Pereira PC, de Magalhaes Lopes CA. Intestinal microbiota of patients with bacterial infection of the respiratory tract treated with amoxicillin. *Brazilian Journal of Infectious Diseases* 2005;9(4):292-300.

38. Pallav K, Dowd SE, Villafuerte J, et al. Effects of polysaccharopeptide from Trametes versicolor and amoxicillin on the gut microbiome of healthy volunteers: a randomized clinical trial. *Gut Microbes* 2014;5(4):458-67. doi: 10.4161/gmic.29558 [published Online First: 2014/07/10]

39. Abeles SR, Jones MB, Santiago-Rodriguez TM, et al. Microbial diversity in individuals and their household contacts following typical antibiotic courses. *Microbiome* 2016;4(1):39. doi: https://dx.doi.org/10.1186/s40168-016-0187-9

40. Mangin I, Suau A, Gotteland M, et al. Amoxicillin treatment modifies the composition of Bifidobacterium species in infant intestinal microbiota. *Anaerobe* 2010;16(4):433-8. doi: 10.1016/j.anaerobe.2010.06.005 [published Online First: 2010/07/06]

41. Christensson B, Nilsson-Ehle I, Ljungberg B, et al. A randomized multicenter trial to compare the influence of cefaclor and amoxycillin on the colonization resistance of the digestive tract in patients with lower respiratory tract infection. *Infection* 1991;19(4):208-15. doi: 10.1007/BF01644947

42. Brismar B, Edlund C, Nord CE. Impact of cefpodoxime proxetil and amoxicillin on the normal oral and intestinal microflora. *European journal of clinical microbiology & infectious diseases : official publication of the European Society of Clinical Microbiology* 1993;12(9):714-9. [published Online First: 1993/09/01]

For peer review only - http://bmjopen.bmj.com/site/about/guidelines.xhtml

43. Edlund C, Stark C, Nord CE. The relationship between an increase in beta-lactamase activity after oral administration of three new cephalosporins and protection against intestinal ecological disturbances. *The Journal of antimicrobial chemotherapy* 1994;34(1):127-38. [published Online First: 1994/07/01]

44. Ladirat SE, Schoterman MH, Rahaoui H, et al. Exploring the effects of galacto-oligosaccharides on the gut microbiota of healthy adults receiving amoxicillin treatment. *British Journal of Nutrition* 2014;112(4):536-46.

45. Floor M, Akkeren FV, Rozenberg-Arska M, et al. Effect of loracarbef and amoxicillin on the oropharyngeal and intestinal microflora of patients with bronchitis. *Scandinavian Journal of Infectious Diseases* 1994;26(2):191-97. doi: 10.3109/00365549409011783

46. Mangin I, Leveque C Fau - Magne F, Magne F Fau - Suau A, et al. Long-term changes in human colonic Bifidobacterium populations induced by a 5-day oral amoxicillin-clavulanic acid treatment. *PLOS ONE* 2012;7(1932-6203 (Electronic)):e50257.

47. Forssten S, Evans M, Wilson D, et al. Influence of a probiotic mixture on antibiotic induced microbiota disturbances. *World Journal of Gastroenterology* 2014;20(33):11878-85.

48. Young VB, Schmidt TM. Antibiotic-associated diarrhea accompanied by large-scale alterations in the composition of the fecal microbiota. *Journal of Clinical Microbiology* 2004;42(3):1203-6.

49. Engelbrektson A, Korzenik JR, Pittler A, et al. Probiotics to minimize the disruption of faecal microbiota in healthy subjects undergoing antibiotic therapy. *Journal of Medical Microbiology* 2009;58(Pt 5):663-70.

For peer review only

For peer review only - http://bmjopen.bmj.com/site/about/guidelines.xhtml

50. Lode H, Von der Hoh N, Ziege S, et al. Ecological effects of linezolid versus amoxicillin/clavulanic acid on the normal intestinal microflora. *Scand J Infect Dis* 2001;33(12):899-903. [published Online First: 2002/03/01]

51. Kabbani TA, Pallav K, Dowd SE, et al. Prospective randomized controlled study on the effects of Saccharomyces boulardii CNCM I-745 and amoxicillin-clavulanate or the combination on the gut microbiota of healthy volunteers. *GUT MICROBES* 2017;8(1949-0984 (Electronic)):17-32.

52. Stewardson AJ, Gaia N, Francois P, et al. Collateral damage from oral ciprofloxacin versus nitrofurantoin in outpatients with urinary tract infections: a culture-free analysis of gut microbiota. *Clinical Microbiology & Infection* 2015;21(4):344.e1-11.

53. Vervoort J, Xavier BB, Stewardson A, et al. Metagenomic analysis of the impact of nitrofurantoin treatment on the human faecal microbiota. *The Journal of antimicrobial chemotherapy* 2015;70(7):1989-92. doi: 10.1093/jac/dkv062 [published Online First: 2015/03/15]

54. Mavromanolakis E, Maraki S, Samonis G, et al. Effect of norfloxacin, trimethoprim-sulfamethoxazole and nitrofurantoin on fecal flora of women with recurrent urinary tract infections. *J CHEMOTHER* 1997;9(3):203-07. doi: 10.1179/joc.1997.9.3.203

55. Heimdahl AF, Nord CE. Influence of doxycycline on the normal human flora and colonization of the oral cavity and colon. *Scand J Infect Dis* 1983;15(0036-5548 (Print)):293-302.

56. Walker C, Preshaw PM, Novak J, et al. Long-term treatment with sub-antimicrobial dose doxycycline has no antibacterial effect on intestinal flora. *Journal of Clinical Periodontology* 2005;32(11):1163-69.

For peer review only - http://bmjopen.bmj.com/site/about/guidelines.xhtml

57. Matto J, Maukonen J, Alakomi HL, et al. Influence of oral doxycycline therapy on the diversity and antibiotic susceptibility of human intestinal bifidobacterial population. *Journal of applied microbiology* 2008;105(1):279-89. doi: 10.1111/j.1365-2672.2008.03792.x [published Online First: 2008/04/10]

58. Rashid MU, Panagiotidis G, Backstrom T, et al. Ecological impact of doxycycline at low dose on normal oropharyngeal and intestinal microflora. *International Journal of Antimicrobial Agents* 2013;41(1872-7913 (Electronic)):352-57.

59. Brismar B, Edlund C, Nord CE. Comparative effects of clarithromycin and erythromycin on the normal intestinal microflora. *Scandinavian Journal of Infectious Diseases* 1991;23(5):635-42. doi: 10.3109/00365549109105189

60. Edlund C, Beyer G, Hiemer-Bau M, et al. Comparative effects of moxifloxacin and clarithromycin on the normal intestinal microflora. *Scandinavian Journal of Infectious Diseases* 2000;32(1):81-85.

61. Edlund C, Alvan G, Barkholt L, et al. Pharmacokinetics and comparative effects of telithromycin (HMR 3647) and clarithromycin on the oropharyngeal and intestinal microflora. *The Journal of antimicrobial chemotherapy* 2000;46(5):741-9. [published Online First: 2000/11/04]

62. Matute AJ, Schurink Ca Fau - Krijnen RMC, Krijnen Rm Fau - Florijn A, et al. Double-blind, placebo-controlled study comparing the effect of azithromycin with clarithromycin on oropharyngeal and bowel microflora in volunteers. *European journal of clinical microbiology & infectious diseases : official publication of the European Society of Clinical Microbiology* 2002;21(0934-9723 (Print)):427-31.

For peer review only - http://bmjopen.bmj.com/site/about/guidelines.xhtml

63. Heimdahl AF, Nord CE. Effect of phenoxymethylpenicillin and clindamycin on the oral, throat and faecal microflora of man. *Scand J Infect Dis* 1979;11(0036-5548 (Print)):233-42.

64. Adamsson I, Edlund C, Sjostedt S, et al. Comparative effects of cefadroxil and phenoxymethylpenicillin on the normal oropharyngeal and intestinal microflora. *Infection* 1997;25(3):154-8. [published Online First: 1997/05/01]

65. Heimdahl AF, Nord CE. Influence of erythromycin on the normal human flora and colonization of the oral cavity, throat and colon. *Scand J Infect Dis* 1982;14(0036-5548 (Print)):49-56.

66. Higgins J, Green S. Cochrane Handbook for Systematic Reviews of Interventions Version 5.1.0 [updated March 2011]. *The Cochrane Collaboration* 2011

67. Eckburg PB, Bik EM, Bernstein CN, et al. Diversity of the human intestinal microbial flora. *Science* 2005;308(5728):1635-8. doi: 10.1126/science.1110591 [published Online First: 2005/04/16]

68. Jernberg C, Löfmark S, Edlund C, et al. Long-term impacts of antibiotic exposure on the human intestinal microbiota. *Microbiology* 2010;156(Pt 11):3216-23. doi: 10.1099/mic.0.040618-0 [published Online First: 2010/08/14]

69. Sullivan Å, Edlund C, Nord CE. Effect of antimicrobial agents on the ecological balance of human microflora. *The Lancet Infectious Diseases* 2001;1(2):101-14. doi: https://doi.org/10.1016/S1473-3099(01)00066-4

70. Zimmermann P, Curtis N. The effect of antibiotics on the composition of the intestinal microbiota - a systematic review. (1532-2742 (Electronic))

71. Hur KY, Lee M-S. Gut Microbiota and Metabolic Disorders. *Diabetes Metab J* 2015;39(3):198-203. doi: 10.4093/dmj.2015.39.3.198

For peer review only - http://bmjopen.bmj.com/site/about/guidelines.xhtml

72. Lazar V, Ditu L-M, Pircalabioru GG, et al. Aspects of Gut Microbiota and Immune System Interactions in Infectious Diseases, Immunopathology, and Cancer. *Frontiers in Immunology* 2018;9:1830.

73. Brugère J-F, Mihajlovski A, Missaoui M, et al. Tools for stools: The challenge of assessing human intestinal microbiota using molecular diagnostics2009.

74. Francino MP, Moya A. EFFECTS OF ANTIBIOTIC USE ON THE MICROBIOTA OF THE GUT AND ASSOCIATED ALTERATIONS OF IMMUNITY AND METABOLISM. *EMJ Gastroenterol* 2013;1:74-80.

75. Sekirov I, Russell SL, Antunes LCM, et al. Gut Microbiota in Health and Disease. *Physiological Reviews* 2010;90(3):859-904. doi: 10.1152/physrev.00045.2009

For peer review only

Figure legends

Figure 1. PRISMA flow chart for study selection.

Figure 2. Overall risk of bias assessment.

Table 1. Search strategy

Table 2. PICOS criteria for inclusion and exclusion of studies.

Table 3. Summary of studies included in the review

Table 4. Impact of Amoxicillin on the gastrointestinal microflora.

Table 5. Impact of Amoxicillin with Clavulanic acid on the gastrointestinal microflora.

For peer review only

For peer review only - http://bmjopen.bmj.com/site/about/guidelines.xhtml

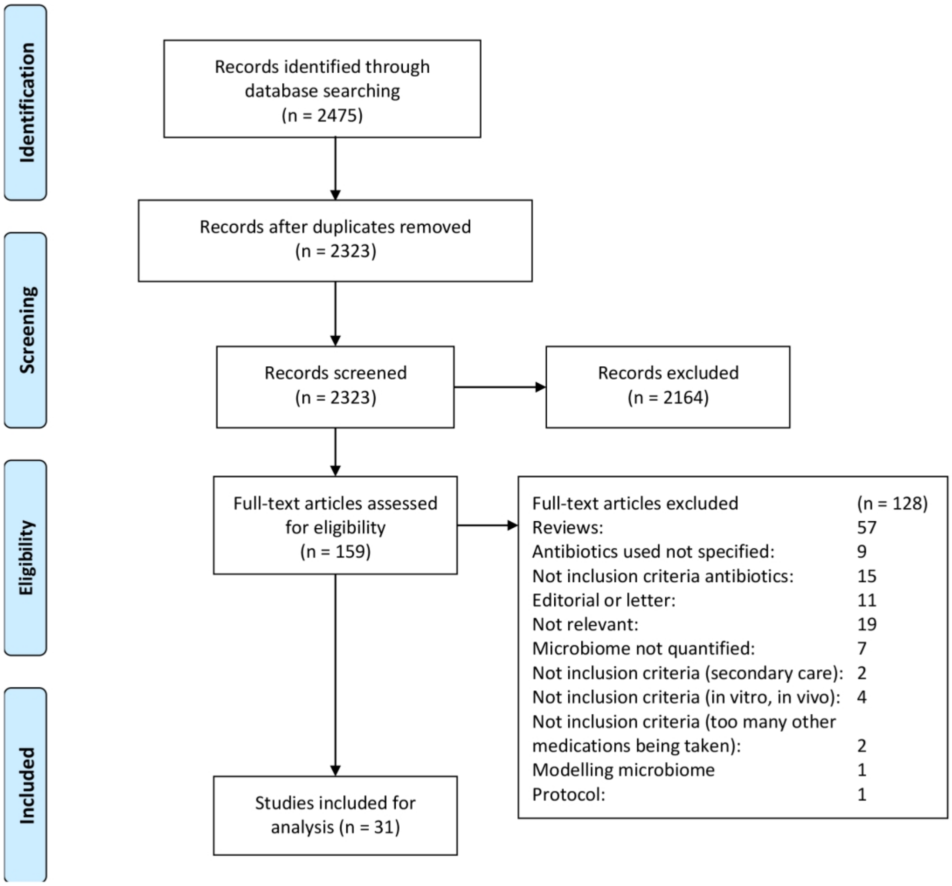

Figure 1. PRISMA flow chart for study selection.

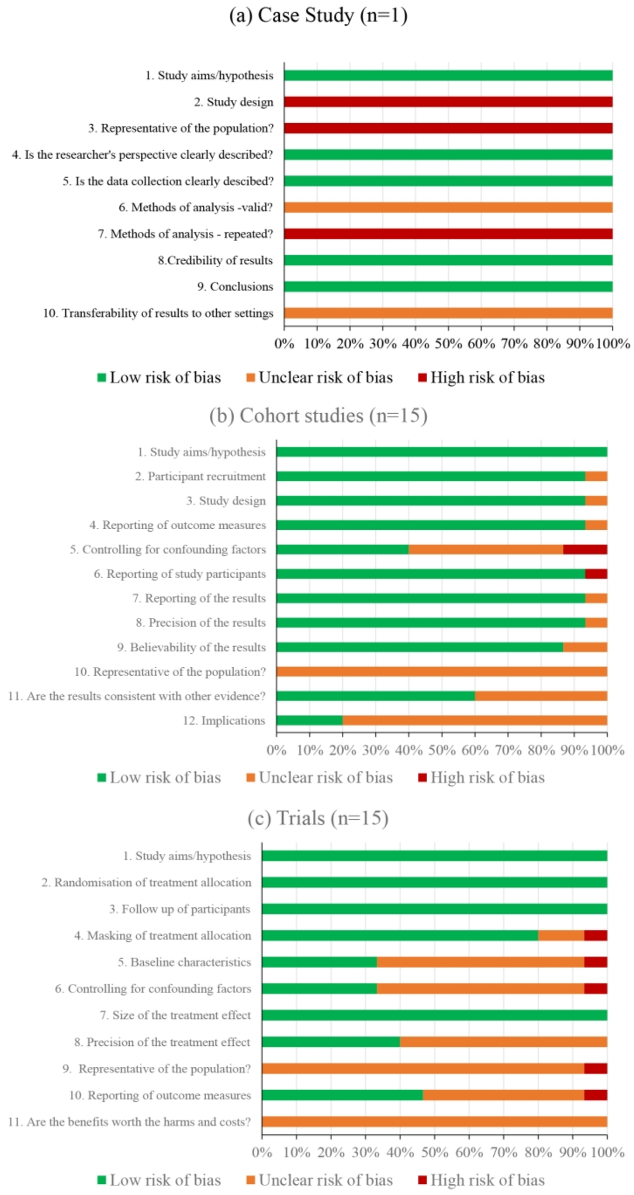

Figure 2. Overall risk of bias assessment.

PRISMA 2009 Checklist

Section/topic	#	Checklist item	Reported on page #
**TITLE**			
Title	1	Identify the report as a systematic review, meta-analysis, or both.	1
**ABSTRACT**			
Structured summary	2	Provide a structured summary including, as applicable: background; objectives; data sources; study eligibility criteria, participants, and interventions; study appraisal and synthesis methods; results; limitations; conclusions and implications of key findings; systematic review registration number.	3-5
**INTRODUCTION**			
Rationale	3	Describe the rationale for the review in the context of what is already known.	6-8
Objectives	4	Provide an explicit statement of questions being addressed with reference to participants, interventions, comparisons, outcomes, and study design (PICOS).	8
**METHODS**			
Protocol and registration	5	Indicate if a review protocol exists, if and where it can be accessed (e.g., Web address), and, if available, provide registration information including registration number.	9
Eligibility criteria	6	Specify study characteristics (e.g., PICOS, length of follow-up) and report characteristics (e.g., years considered, language, publication status) used as criteria for eligibility, giving rationale.	10-11; Table 2
Information sources	7	Describe all information sources (e.g., databases with dates of coverage, contact with study authors to identify additional studies) in the search and date last searched.	9
Search	8	Present full electronic search strategy for at least one database, including any limits used, such that it could be repeated.	9-10 Table 1
Study selection	9	State the process for selecting studies (i.e., screening, eligibility, included in systematic review, and, if applicable, included in the meta-analysis).	10
Data collection process	10	Describe method of data extraction from reports (e.g., piloted forms, independently, in duplicate) and any processes for obtaining and confirming data from investigators.	11-12
Data items	11	List and define all variables for which data were sought (e.g., PICOS, funding sources) and any assumptions and simplifications made.	12
Risk of bias in individual studies	12	Describe methods used for assessing risk of bias of individual studies (including specification of whether this was done at the study or outcome level), and how this information is to be used in any data synthesis.	33
Summary measures	13	State the principal summary measures (e.g., risk ratio, difference in means).	33
Synthesis of results	14	Describe the methods of handling data and combining results of studies, if done, including measures of consistency (e.g., I^2) for each meta-analysis.	33

For peer review only - http://bmjopen.bmj.com/site/about/guidelines.xhtml

PRISMA 2009 Checklist

Section/topic	#	Checklist item	Reported on page #
Risk of bias across studies	15	Specify any assessment of risk of bias that may affect the cumulative evidence (e.g., publication bias, selective reporting within studies).	33
Additional analyses	16	Describe methods of additional analyses (e.g., sensitivity or subgroup analyses, meta-regression), if done, indicating which were pre-specified.	Not applicable
**RESULTS**			
Study selection	17	Give numbers of studies screened, assessed for eligibility, and included in the review, with reasons for exclusions at each stage, ideally with a flow diagram.	34; Figure 1
Study characteristics	18	For each study, present characteristics for which data were extracted (e.g., study size, PICOS, follow-up period) and provide the citations.	13-32 Table 3
Risk of bias within studies	19	Present data on risk of bias of each study and, if available, any outcome level assessment (see item 12).	35-37; Figure 2
Results of individual studies	20	For all outcomes considered (benefits or harms), present, for each study: (a) simple summary data for each intervention group (b) effect estimates and confidence intervals, ideally with a forest plot.	37-53 Table 3 & 4
Synthesis of results	21	Present results of each meta-analysis done, including confidence intervals and measures of consistency.	Not applicable
Risk of bias across studies	22	Present results of any assessment of risk of bias across studies (see Item 15).	Not applicable
Additional analysis	23	Give results of additional analyses, if done (e.g., sensitivity or subgroup analyses, meta-regression [see Item 16]).	Not applicable
**DISCUSSION**			
Summary of evidence	24	Summarize the main findings including the strength of evidence for each main outcome; consider their relevance to key groups (e.g., healthcare providers, users, and policy makers).	53-56
Limitations	25	Discuss limitations at study and outcome level (e.g., risk of bias), and at review-level (e.g., incomplete retrieval of identified research, reporting bias).	56-57
Conclusions	26	Provide a general interpretation of the results in the context of other evidence, and implications for future research.	57-58
**FUNDING**			
Funding	27	Describe sources of funding for the systematic review and other support (e.g., supply of data); role of funders for the systematic review.	58

From: Moher D, Liberati A, Tetzlaff J, Altman DG, The PRISMA Group (2009). Preferred Reporting Items for Systematic Reviews and Meta-Analyses: The PRISMA Statement. PLoS Med 6(7): e1000097. doi:10.1371/journal.pmed1000097

For more information, visit: **www.prisma-statement.org**.

For peer review only - http://bmjopen.bmj.com/site/about/guidelines.xhtml
